# Cultural Responses to Covid-19 Pandemic: Religions, Illness Perception, and Perceived Stress

**DOI:** 10.3389/fpsyg.2021.634863

**Published:** 2021-07-23

**Authors:** Rachel Sing-Kiat Ting, Yue-Yun Aw Yong, Min-Min Tan, Chee-Khong Yap

**Affiliations:** ^1^Department of Psychology, Jeffrey Cheah School of Medicine and Health Sciences, Monash University Malaysia, Subang Jaya, Malaysia; ^2^South East Asia Community Observatory, Jeffrey Cheah School of Medicine and Health Sciences, Monash University Malaysia, Segamat, Malaysia; ^3^Sunway Medical Centre, Subang Jaya, Malaysia

**Keywords:** Covid-19, stress, religions, culture, illness perception, religious expression

## Abstract

Many psychological researchers have proven the deteriorating effects of the coronavirus disease 2019 (Covid-19) pandemic on public mental health. In Malaysia, various Covid-19 clusters were associated with religious gatherings. From a cultural psychology perspective, how ethno-religious groups respond to this crisis originating from their unique rationality and ecological systems. Therefore, this study aimed to explore the illness perceptions of major religious groups (Christian, Muslim, and Buddhist) in Malaysia toward the Covid-19 pandemic, their stress levels, and the relationship between illness perception, stress, and forms of religious expression during the lockdown period. Through an online survey method, 608 Malaysian religious believers were included in this mixed-method empirical study, which adapted standardized instruments [Duke University Religion Index (DUREL), Brief Illness Perception Questionnaire (BIPQ), and Perceived Stress Scale (PSS)]. Statistical analysis showed that all three groups reported moderate levels of stress in average without any significant difference after controlling for age. Both internal and external forms of religious expression had a significant negative relationship with stress levels. Personal control, comprehension, and emotions domains of illness perception accounted for a significant variance in the stress level. Furthermore, religious expression significantly moderated the relationship between some illness perception domains and stress. Qualitative coding revealed that most participants perceived human behavior and attitudes, sociopolitical, and sociological factors as causal factors to the current pandemic. These findings confirmed the relationship between religious expression, illness belief, and stress regulation during the pandemic lockdown. Incidental findings of age as a potential protective factor for Malaysian believers warrants further study. In the conclusion, implications for public health policymakers and religious communities on pandemic prevention and well-being promotion were discussed.

## Introduction

### Covid-19 Pandemic Impact on Mental Health in Malaysia

Since its outbreak from China in December 2019, the coronavirus disease 2019 (Covid-19) has evolved rapidly into a global pandemic. As of June 4, 2021, approximately 595,374 confirmed Covid-19 cases had been reported in Malaysia, with 3,096 deaths; and its infection rate have surpassed India and United States, and it tops ASEAN in daily new Covid-19 deaths per capita (Roser et al., 2021; World Health Organization [WHO], 2020b). The rapid spread of Covid-19 resulted in the Malaysian government imposing the first movement control order (MCO), a nationwide lockdown strategy, first on March 18, 2020 (Minhat and Shahar, 2020; The Sun Daily, 2020) to combat the spread of the virus (Lippi et al., 2020). Despite initially successful with restrictions lifted in July 2020, Malaysia entered into a total lockdown once again as the pandemic exponentially escalated in April 2021 (World Health Organization [WHO], 2020a; Channel News Asia, 2021; Rodzi, 2021).

People worldwide are faced with significant health, economic, and social challenges exacerbated during the current global pandemic (Chakraborty and Maity, 2020). Concerns have risen in Malaysia on the adverse effects of the Covid-19 pandemic and prolonged MCO on the mental health of vulnerable populations resulting from social isolation, the loss of income, and an exposure to toxic family environments (Shanmugam et al., 2020; Yusof, 2021). Rising trends of depression and anxiety, increased cases of reported domestic violence, marital distress, and a disproportionate spike in suicide rates and stress levels during MCO have been reported by local media (Abdullah, 2020; Dorall, 2020; The Star, 2020a; Togoh, 2020). About 85.5% of the government-run Covid-19 hotline calls in 2021 were for mental health support, with many citing extreme stress caused by financial, relationship, and mental health struggles (Aziz et al., 2020; Hassan, 2020; Malay Mail, 2021). Since stress is a strong predictor of depression, anxiety, and other mental health disorders (Marin et al., 2011; Guo et al., 2020; Montano and Acebes, 2020), the search for cultural resilience in the face of inevitable stress caused by the pandemic and ongoing lockdowns is needed.

Amidst these extraneous circumstances, culture and religion appear to play a paradoxical role in shaping the communal cognition (“Why it happened?”) and responses (“What should we do about it?”) toward Covid-19. Historically, humans have faced various contagious diseases such as the plague (The Black Death), AIDS, severe acute respiratory syndrome (SARS), Ebola, and now Covid-19. When faced with a pandemic, people naturally demand and seek explanations as a response to their vulnerabilities (Rosenberg, 1992). It is expected that different cultural systems have differing sets of illness perceptions activated during a pandemic crisis, leading to varying stress coping mechanisms. However, due to the nature of Covid-19 transmission pathways and the need for the religious faithful to congregate, global religious communities experienced infection in the early phase of the pandemic, leading to a polarized view between “religion as a cure” vs. “religion as a curse.” In Malaysia, about 48% of the positive Covid-19 cases in 2020 were linked to a large international Islamic event that took place from February 27, 2020 to March 1, 2020 at Kuala Lumpur attended by about 16,000 devotees. Another large Covid-19 cluster began with a Christian event held around the same time (Tan et al., 2021). Through examining illness perception in the landscape of cultural psychology, a less biased approach toward the display of religious behaviors by the public during a pandemic (even if it contradicts modern medical knowledge) can be achieved. This is especially important in a multicultural and multireligious nation like Malaysia.

### Religion, Well-Being, and Illness Perceptions

#### Culture and Religious Expression

In the postmodern era, “culture” is no longer defined by ethnicity, geography, nationality, or any skin color group, but by the unique resources available for humans to make sense of their world (Rein, 2016) or the adaptive ecosystem (Tucker, 2013). According to Saroglou and Cohen (2011), the relationship between culture and religion could be conceptualized *via* the following six frameworks—religion as a part of culture, religion constitutes culture, religion includes and transcends culture, religion influenced by culture, religion shapes culture, and religion interacts with culture in influencing cognitions, emotions, and actions. In this study, we adopt the last aforementioned framework with an added perspective of evolutionary psychology that, religion co-evolves with human cognition, giving form to a dynamic cultural system that embodies a unique epistemology of illnesses and healing (Belzen, 2010; Ting and Sundararajan, 2018; Dueck, 2020). In the past, various dimensions of religion have been used to operationalize religiosity, such as the frequency of church attendance (organizational religiosity), private religious activities (non-organizational religiosity), intrinsic beliefs (intrinsic religiosity), religious importance, and religious experiences (Hood et al., 2018). However, many religiosity measurements were developed based on the Judeo-Christianity faith in the Western society, failing to capture the full spectrum of diversity in religious expression in Asian societies (Hill and Pargament, 2003; Hill and Edwards, 2013; Ting et al., 2019; Dueck, 2020).

Based on Granovetter (1973) network theory, there are two fundamental social networking types—strong- and weak-tie-based relationships. Expanding on network theory and evolutionary sciences (Todd and Gigerenzer, 2012), Sundararajan (2015, 2020) proposed a culture-cognition scheme—the ecological rationality framework, where (a) strong-ties societies, referring to communities with lower relational mobility and based on small, intimate connections with kinship and close friends would adopt a more holistic mode of rationalities, thus orienting them to the external environment and (b) weak-ties societies referring to communities with higher relational unfamiliar or mobility that rely on cooperation with unrelated others, engaging in networking and association with acquaintances and strangers. Weak-ties societies privilege an analytic mode of rationality, thus orienting them to the internal mental space. This observation is similar to the cognitive style differences found between Westerner and Asians by Nisbett and Miyamoto (2005), and later by Talhelm and English (2020) on southern vs. northern China due to agricultural ecology. The division of strong- vs. weak-ties rationality has also been found among diverse religious communities for Yi ethnic minority in China in a series of study by Ting and Sundararajan’s research team (Ting et al., 2017, 2020; Ting and Sundararajan, 2018).

Applying the ecological rationality framework (ERF) model, this study proposed that the taxonomy of religiosity (a form of rationality) found in two major cultural ecologies—strong- vs. weak-ties, could be framed as external vs. internal religious (ER vs. IR) expressions. In this case, strong-ties society like Malaysia would capitalize on externally oriented religious expression in social space (e.g., rituals and ceremonies with social gathering) compared to a weak-ties society that would privilege internally oriented religious expression in private space (e.g., praying and meditation alone). Inferring upon this rationale, inaccessibility to communal practices (e.g., religious rituals) during lockdown is expected to create stress for all religious groups, and might transform the practice of believers for religion from ER to IR. We are curious whether different religious groups in Malaysia adopt different forms of ER vs. IR, and how a different form of religiosity associates with the psychological distress experienced by the believers during the lockdown. Nevertheless, to date, this relationship between religious expression and Covid-19 stress has yet to be empirically tested.

#### Religious Coping and Mental Well-Being

The influence of religion on one’s psychological processes might affect one’s health perceptions and coping behaviors (Milstein et al., 2019). A plethora of evidence suggested that religious coping was positively associated with mental health outcomes (Pargament, 1997; Pargament et al., 2000; Weber and Pargament, 2014; Khodaveirdyzadeh et al., 2016; Oman and Syme, 2018), and benefitted patients in terms of quality of life, sense of meaning, mental health, acceptance, source of comfort, and hope (Roger and Hatala, 2017). Similar benefits of religious coping have been found in some Malaysian studies. A case study conducted by Ting and Ng (2012) showed that the incorporation of spiritual resources in psychotherapy was significantly beneficial and socially acceptable by the Chinese in Malaysia (CIM) community, thus reducing the stigma associated with seeking psychological help. A few studies on Muslims in Malaysia (Shaw et al., 2018; Ahmadi et al., 2019) highlighted that religiosity and spirituality play a significant role in their health beliefs and health behaviors. Additionally, a study on religion and mental health among CIM older adults by Tan et al. (2020) showed that belief in a higher power was negatively associated with psychological distress, indicating that religious beliefs could be an essential resource in helping Malaysians to cope with life stressors. However, the past studies did not further differentiate between ER or IR expressions espoused by the Malaysian religious believers and whether religious coping would be helpful in curbing pandemic-related stress.

#### Religion, Illness Perception, and Stress

According to the self-regulatory model proposed by Leventhal et al. (1998), illness perception is a construct that describes how an individual perceives his or her disease in domains such as identity (the meaning of symptoms and disease), timeline (the development and chronicity of the illness), consequences (perceived or real impact of the illness), control (outcome expectancy and sense of control in managing the illness), and causes (attributions of the illness). It involves two routes of mental processing—cognitive and emotional representations. The presence of contextual stimuli (e.g., Covid-19 pandemic) creates both cognitive and emotional representations of the illness, thus forming an illness perception that then informs the adoption of differing coping responses, leading to different emotional and health outcomes. It was found that illness perceptions (i.e., consequences, timeline, personal control, treatment control, identity, concern, and emotional response) were all significantly correlated with anxiety and depression (Zhang et al., 2016). Specifically, consequences, an understanding of the disease (comprehension), emotional representation, and the experience of symptoms (identity), may predict perceived stress (Miceli et al., 2019). According to the model, coping strategies serve as the self-regulated pathway between illness representation and mental health outcomes. Therefore, it is inferred that religious coping might also moderate the stress caused by the illness perception toward Covid-19 pandemic, which is one of the aims in our study.

On the other hand, illness perception is also heavily influenced by cultural factors, such as religious beliefs. For example, in a Malaysian cultural context, influenced by the perspective of traditional Chinese medicine, general Chinese-Taoist perceive the disease as an imbalance of forces within the body system (Chew et al., 2011) and rural Chinese attributed stroke to poor blood flow due to “wind” blocking and thick blood (Yap et al., 2019). On the other hand, Malay-Muslims believe that illnesses and suffering are trials from God by which one’s sins are removed and are a part of one’s life journey to an everlasting world (Attum et al., 2020). Illnesses were perceived as opportunities for spiritual growth and rewards (Al-Khayat, 2004). For Buddhist in Malaysia, the beliefs of karma and reincarnation lead to the perception that illnesses and sufferings are the results of sin in one’s past life by the believers (Ahmad, 2007; Tang, 2015; Samuels, 2017). Similar to the global Christian community, Christians in Malaysia generally adhere to the religious belief that suffering, including illnesses can be caused by personal sin, testing from God, weakening of faith, and punishment from God (Ting and Watson, 2007). A recent systematic review of pandemic perceptions (Yap et al., 2021) also found that different religious traditions hold differing beliefs regarding infectious disease for epidemic like AIDS transmission. However, Covid-19 pandemic perceptions among different religious groups remain understudied to date. Hence, this study will adopt an exploratory stance to solicit the attribution of pandemic across all three religious groups.

#### Research Aims and Questions

Of the 32.6 million population in Malaysia, 69.3% are Bumiputras (natives), which consist of Malays and a minority of indigenous people, 22.8% Chinese, 6.9% Indians, and 1.0% other races (Department of Statistics Malaysia, 2011). Islam is the official religion of Malaysia and practiced by 61.3% of the population (Department of Statistics Malaysia, 2011). Legally, all Malays are Muslims, which reflects the intersection of ethnic identity and religious identity among the Muslim group. The second largest religion practiced in Malaysia is Buddhism (19.8%), followed by Christianity (9.2%) and Hinduism (6.3%). The majority of Chinese (83.6%) and Indians (86.2%) are Buddhists and Hindus, respectively, demonstrating a high overlap between religion and ethnicity in Malaysia. This unique multicultural landscape requires a unique cultural perspective toward ethno-religion, rather than separating ethnicity and religion as two different concepts.

**FIGURE 1:**
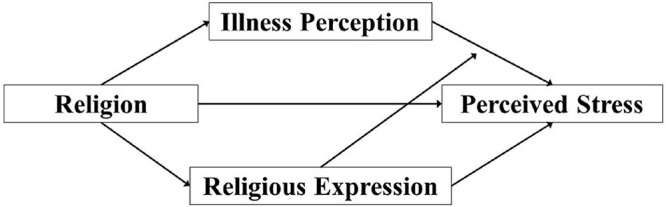
Theoretical framework of the relationship between religion, illness perception, religious expression, and perceived stress.

Covid-19 perceptions espoused by Malaysians are yet to be identified to date, especially across the various ethno-religious groups. In addition, though religiosity as a variable had often been associated with positive health outcomes, its multifaceted expression had not been fully examined in relation to stress regulation during pandemic. Therefore, this study aimed to explore: (a) religious believers’ level of stress, and their perceptions toward the Covid-19 outbreak, (b) how such perceptions affect stress levels, and (c) how different forms of religious expression moderate stress levels caused by illness perception during the lockdown in Malaysia. There are two parts in our conceptual framework (Figure 1)—in the first part, since different religious groups represent unique ecological systems, there would be differences in their perceptions of Covid-19, their forms of religious expression, and perceived stress level; in the second part, according to religious coping theory and self-regulatory theory, religiosity could play a role in reducing the stress caused by the illness perception.

##### Research question 1

Are there differences in perceived stress, religious expressions, and illness perceptions between different religious groups?

Hypothesis 1a: Due to the lockdown, we hypothesized that there would be no differences in the perceived stress levels of the three religious groups.

Hypothesis 1b: Due to the exploratory nature, we hypothesized that there would be differences in the illness perception domains of the three religious groups without a specific direction.

Hypothesis 1c: Due to the exploratory nature, we hypothesized that there would be differences in the religious expression domains of the three religious groups without a specific direction.

##### Research questions 2

What is the relationship between religious expression, illness perceptions, and perceived stress levels?

Hypothesis 2a: According to Religious coping theory, there would be a significant negative relationship between religious expression and perceived stress.

Hypothesis 2b: According to self-regulatory model, there would be a significant positive relationship between certain illness perception domains (consequences, timeline, concern, emotions, severity, and likelihood of contracting) and a negative relationship between certain illness perception domains (personal control and comprehensibility) with perceived stress.

Hypothesis 2c: According to self-regulatory model, the relationship between illness perception domains and perceived stress would be moderated by religious expression, with ER enhancing the stress, and IR reducing the stress.

##### Research question 3

What are the causal attributions of the Covid-19 pandemic across different religious groups?

The first two research questions were answered *via* quantitative standardized survey questions, while the last question was answered through qualitative coding of textual responses to an open-ended question embedded in the survey.

## Methodology

### Design and Procedure

This study used a mixed-methods research design with cross-sectional data, and was part of a larger national research project. After providing informed consent, participants completed a 10–15 min online survey, which included quantitative and qualitative questions. Explanatory statements and informed consent forms were delinked from the online survey to ensure the anonymity of the respondents. As an incentive of participation, ten lucky draws to win a RM50 (USD 12) e-wallet credit was offered. Ethics approval was obtained from the author’s institute before the commencement of the study.

### Participants

Participants were recruited online during the MCO in April–July 2020 *via* emails and social networking sites through voluntary purposive sampling methods. Each potential participant was invited to share and forward the link to the survey to their family and friends who meet the inclusion criteria: (1) Malaysian citizens residing in Malaysia during MCO and (2) aged 18 years and above. *A priori* power analysis using G^∗^Power 3.1 software was performed for sample size estimation (Faul et al., 2009). To detect a medium effect size of *f*^2^ = 0.15 with 80% power (*α* = 0.05, two-tailed) for regression analysis with 12 predictors, a minimum sample size of 127 would be needed (Cohen, 2013).

In total, 738 participants filled up the online survey, including non-religious Malaysians as setting “religion” as an exclusion criterion would be culturally inappropriate. After the removal of duplicates, invalid data, and non-religious participants, 608 participants who identified themselves as religiously affiliated with one of the three major religions were retained (Table 1). Four participants were further excluded from quantitative analysis due to invalid or missing data. Details for participants’ sociodemographic characteristics, religious expression, illness perception, and perceived stress scores were shown in Table 1. The majority of Buddhist (99.17%) and Christian (91.08%) participants were of Chinese ethnicity, whereas the Muslim participants were all of Malay ethnicity. The total percentage of Chinese participants was 80.30%, followed by Malays (14.90%), Indians (1.16%), and others (11.94%). Gender distribution was slightly skewed toward a women majority (total 64.40% women) but was relatively equal across the three religious groups (*p* = 0.136). Christian group have a higher mean of age (*M*_age_ = 39.20, *SD*_age_ = 12.45) than Buddhist (*M*_age_ = 30.31, *SD*_age_ = 10.43) and Muslim groups (*M*_age_ = 33.03, *SD*_age_ = 9.31). Most of the participants were of undergraduate degree education level and were urban or city dwellers. On average, data were collected 29.54 days following the implementation of the lockdown. An ANOVA and the chi-squared test of independence was conducted on sociodemographic variables revealing significant religious group differences in age and education.

**TABLE 1:** Sociodemographic background and descriptive statistics of participants from different religious groups.

	**Buddhists *M (SD)/N (%)***	**Christians *M (SD)/N (%)***	**Muslims *M (SD)/N (%)***	**Total *M (SD)/N (%)***	**ANOVA/**χ^2^**significance value**
**Total**	241 (100)	269 (100)	94 (100)	604 (100.0)	
**Gender**					0.136
Female	150 (62.24)	171 (63.20)	69 (73.40)	389 (64.40)	
Male	91 (37.76)	99 (36.80)	25 (26.60)	215 (35.60)	
**Ethnicity**					
Chinese	239 (99.17)	245 (91.08)	1 (1.06)	485 (80.30)	
Malay	0	0	90 (95.74)	90 (14.90)	
Indian	0	7 (2.60)	0	7 (1.16)	
Others	2 (0.83)	17 (6.32)	3 (3.20)	22 (3.64)	
**Age**	30.31 (10.43)	39.20 (12.45)	33.03 (9.31)	34.70 (11.94)	<0.001
**Education**					<0.001
High school and below	11 (4.56)	45 (16.73)	9 (9.57)	65 (10.76)	
Undergraduate	177 (73.44)	158 (58.74)	59 (62.77)	394 (65.23)	
Postgraduate	53 (21.99)	66 (24.54)	26 (27.66)	145 (24.01)	
**Hometown area**					
Rural	37 (15.35)	28 (10.41)	25 (26.60)	90 (14.90)	
Suburban	64 (26.56)	57 (21.19)	28 (29.79)	149 (24.67)	
Urban	140 (58.09)	185 (68.40)	41 (43.62)	365 (60.43)	
**Current area of residence**					
Rural	13 (5.39)	8 (2.97)	15 (15.96)	36 (5.96)	
Suburban	49 (20.33)	54 (20.07)	23 (24.47)	126 (20.86)	
Urban/City	179 (74.27)	208 (76.95)	56 (59.57)	443 (73.18)	
**Days since MCO^a^**	28.48 (18.94)	30.26 (11.29)	30.19 (16.27)	29.54 (15.52)	0.391
**Religious expression**					
External religious expression	2.38 (0.82)	4.20 (1.00)	3.61 (1.01)	3.38 (1.26)	<0.001
Internal religious expression	3.07 (0.95)	4.60 (0.73)	4.52 (0.90)	3.98 (1.13)	<0.001
**Illness perception**					
1. (*Consequences*) How much does COVID-19 affect your life?	7.11 (2.05)	6.74 (2.33)	6.28 (2.49)	6.82 (2.26)	0.008
2. (*Timeline*) How long do you think the COVID-19 pandemic will continue?	6.83 (1.66)	6.53 (1.67)	6.48 (1.68)	6.64 (1.67)	0.072
3. (*Personal control*) How much control do you feel you have over the COVID-19 pandemic?	5.02 (2.45)	5.00 (2.43)	5.49 (2.54)	5.08 (2.46)	.0206
4. (*Concern*) How concerned are you about the COVID-19 pandemic?	7.62 (1.93)	7.65 (1.98)	7.63 (2.35)	7.64 (2.02)	0.978
5. (*Comprehensibility*) How well do you feel you understand the COVID-19 pandemic?	7.25 (1.48)	7.35 (1.70)	7.97 (1.41)	7.41 (1.59)	0.001
6. (*Emotions*) How much does the COVID-19 pandemic affect you emotionally (e.g., does it make you angry, scared, upset or depressed)?	5.35 (2.50)	4.61 (2.66)	5.47 (2.44)	5.04 (2.59)	0.001
7. (*Severity*) How severe do you think of the COVID-19 as a disease?	8.54 (1.74)	8.56 (1.65)	8.85 (1.55)	8.60 (1.67)	0.272
8. (*Likelihood of contracting*) How likely do you think you would contract the COVID-19?	4.48 (2.18)	4.32 (2.23)	4.72 (2.41)	4.45 (2.24)	0.312
**Perceived stress**	20.27 (6.34)	17.63 (6.93)	18.35 (6.36)	18.79 (6.71)	<0.001
Low perceived stress	37 (15.4)	79 (29.4)	20 (21.3)	136 (22.5)	
Moderate perceived stress	165 (68.5)	162 (60.2)	65 (69.1)	392 (64.9)	
High perceived stress	39 (16.2)	28 (10.4)	9 (9.6%)	76 (12.6)	

*^*a*^The average number of days since movement control restriction was implemented (March 18, 2020) when the participant completed the survey.*

### Measures

All online survey items were provided in English, Mandarin, and Malay, which are common languages among Malaysians. The items were translated by a trilingual research team and backtranslated by Mandarin and Malay native speakers with psychology-related academic qualifications. The survey consisted of the following sections.

#### Demographics

Participants were asked about their age, gender, ethnicity, area of residence, highest education level, language proficiency, and religious affiliation in this section.

#### Religious Expression

To measure religious expression, the Duke University Religion Index (DUREL; Koenig et al., 1997) was adopted and adapted to the Malaysian context (A-DUREL). DUREL is made up of five items developed originally to measure three independent religiosity subtypes: *organizational religiosity* (religious attendance; item one), *non-organizational religiosity* (private religious activities; item two), and *intrinsic religiosity* (items three–five; Koenig et al., 1997). In this study, the A-DUREL items were rephrased to encompass different religious groups, such as the addition of “mosques,” “temples,” “incense burning,” and the replacement of “Bible study” to “reading Holy Scriptures.” To capture folk religious practice in Malaysia, this study included an additional item (*community religious practices*), which evaluated the importance of performing religious ceremonies in the community.

Participants were asked to indicate frequencies of religious activities on item one and two on a five-point scale (1 = *Once a year or less*; 2 = *A few times a year*; 3 = *A few times a month*; 4 = *Once a week*; and 5 = *More than once per week*). For the rest of the items, participants were asked to indicate their agreement on a five-point Likert scale from one (*definitely not true of me*) to five (*definitely true of me*). For the purpose of this study, ER expression was operationally defined by items one (“How often do you attend prayer/worship at temples/church or other religious meetings before MCO?”) and six [“I believe practicing religious rituals together with my family and close community (e.g., attending mass, praying in the religious spaces, burning incense, and burning paper money) is very important”], while the IR expression subscale was operationally defined *via* items two–five (“How often do you spend time in private religious activities, such as praying, meditation, incense burning, or reading Holy Scriptures?,” “In my life, I experience the presence of God or a Supreme Being,” “My religious beliefs are what really lie behind my whole approach to life,” and “I try hard to carry my religion over into all other dealings in life”).

Previous reliability studies on Duke University Religion Index in Malaysia and other countries showed high internal consistency (*α* = 0.78–0.91; Koenig and Büssing, 2010; Nurasikin et al., 2013; Chong et al., 2019). The overall Cronbach’s alpha of A-DUREL’s in this study was 0.90. Cronbach’s alpha for external and IR expression subscales were 0.70 and 0.88, respectively.

#### Illness Representations

Illness representations were measured using the Brief Illness Perception Questionnaire (BIPQ; Broadbent et al., 2006). BIPQ consisted of nine single-item domains assessing perception and beliefs about illness. This study adapted BIPQ (A-BIPQ) to the current pandemic context by (1) replacing “illness” with “Covid-19 pandemic” and (2) replacing the original BIPQ domains treatment control and identity with the severity and likelihood of contracting, respectively. In the A-BIPQ, cognitive representations were assessed with five domains: consequences (item one: “How much does the Covid-19 pandemic affect your life?”), timeline (item two: “How long do you think the Covid-19 pandemic will continue?”), personal control (item three: “How much control do you feel you have over the Covid-19 pandemic?”), severity (item seven: “How severe do you think of Covid-19 as a disease?”), and the likelihood of contracting (item eight: “How likely do you think you would contract the Covid-19?”). Emotional representations were assessed with two items: concern (item four: “How concerned are you about the Covid-19 pandemic?”) and emotions [item six: “How much does the Covid-19 pandemic affect you emotionally (e.g., does it make you angry, scared, upset, or depressed)?”]. Covid-19 comprehensibility was assessed with item five (“How well do you feel you understand the Covid-19 pandemic?”). Causal representation was assessed using one open-ended qualitative response item, where participants were asked to list the three most important causal factors of Covid-19 (item nine: “Please list in rank order the three most important factors that you believe caused the Covid-19 pandemic”).

The BIPQ developed by Broadbent et al. (2006) was derived from the established Illness Perception Questionnaire (IPQ), which involves a lengthy administration time and higher costs. The single-item format of BIPQ has been tested for test–retest reliability, concurrent validity, discriminant validity, and predictive validity across various studies and contexts worldwide, including Malaysia. In spite of utilizing a single-item measurement, the brevity of BIPQ guarantees a higher response rate, prevents survey fatigue, and encourages participation. It has also been shown to have good concurrent validity with IPQ (Broadbent et al., 2006). All A-BIPQ items were scored on a 0–10 response scale, with higher scores indicating stronger beliefs. Past studies have shown good test–retest reliability (*r* = 0.39–0.78) in Western and Malaysian populations, and good validity (Broadbent et al., 2006; Chew et al., 2017).

#### Perceived Stress

Perceived stress was measured using the Perceived Stress Scale-10 (PSS-10), and a 10-item self-reported questionnaire was widely used to measure the perceived stress levels of an individual “in the last month” (Cohen and Williamson, 1988; Taylor, 2015). For this study, the phrase “in the last month” was replaced with “during the Covid-19 outbreak” to capture perceived stress during the pandemic. Participants were asked to indicate how frequently certain thoughts and feelings occurred during the pandemic by rating on a five-point Likert’s scale from 0 (*never*) to 4 (*very often*). There were four positive and six negative stress perception items. Examples of positive and negative stress perception items are “During the Covid-19 outbreak, how often have you felt that you were on the top of things?” and “During the Covid-19 outbreak, how often have you felt that you were unable to control the important things in your life?,” respectively. Total PSS scores were obtained by summing the ten items after reverse- coding of four positive stress perception items (items four, five, seven, and eight). Higher scores indicate higher perceived stress levels (low = 0–13, moderate = 14–26, high = 27–40). Multiple studies have provided support for the construct and concurrent validity and reliability (*α* = 0.83–0.89) of PSS-10 in various settings and populations (Roberti et al., 2006; Leung et al., 2010). Cronbach’s alpha for this study was 0.86.

### Data Analysis

#### Quantitative Data Analysis

Data were cleaned and analyzed using SPSS (v26; IBM Corp, 2017) and R statistical software (R Core Team, 2013). ANOVA and analysis of covariance (ANCOVA) were performed to test for religious group differences on perceived stress, illness perception, and religious expression. *Post hoc* analyses with 98% Bonferroni correction were conducted for variables that were significant in ANOVA and ANCOVA. One-sample *t*-tests were conducted to assess if there were significant differences between the ER and IR expression for each religious group. Bivariate correlations were conducted to assess the relationship between perceived stress, illness perception, and religious expression. Lastly, multiple regression and moderated multiple regression analyses were conducted to further clarify the relationships between perceived stress, illness perception, and religious expression.

#### Qualitative Data Analysis

A causal representation of pandemic was assessed using one open-ended qualitative response item, where participants were asked to list the three most important causal factors of Covid-19 (BIPQ item nine). Textual data were coded by the research team using an inductive-deductive thematic analysis approach (Braun and Clarke, 2006; Fereday and Muir-Cochrane, 2006). The process of coding was based on Braun and Clarke (2006) thematic analysis guidelines and Morse’s (2015) rigor in qualitative inquiry criteria, as follows:

(1) Data were organized and placed in a spreadsheet with multiple rows and columns to form an overall data table (Vaughn and Turner, 2016). Data were read several times (repeated reading technique; Braun and Clarke, 2006) thoroughly by two independent coders for familiarization.
(2) Coding was performed manually. First-level codes were generated systematically based on the responses of participants. Similar responses were categorized into first-level codes. At this phase, data were not interpreted. Each response was attended to with full and equal attention.
(3) First-level codes were analyzed and combined to identify subthemes (second-level coding). Subthemes were then subsequently collated into overarching themes (third-level coding) inductively and deductively. As the coding process was spanned over weeks, a qualitative codebook was developed to ensure consistency across large amounts of data (Morse, 2015).
(4) Data were also reviewed and independently coded by two researchers and the principal investigator as the auditor for internal consistency. Discrepancies were resolved by consensus discussion between the two coders and the auditor (Morse, 2015). All codes and themes were subsequently reviewed and refined by assessing their internal homogeneity and external heterogeneity (Patton, 1990). Internal homogeneity indicated that the data within a theme should be cohesive and meaningful while external heterogeneity indicated that the data within a theme should differ and are identifiable from other themes (Braun and Clarke, 2006).
(5) Percentage weightage was then tabulated based on the frequency of responses in each theme and subthemes across the three religious groups.

## Results

### Quantitative Analysis

Hypothesis 1a: There would be no differences in the perceived stress levels of the three religious groups.

Descriptive analysis showed that all three religious groups experience moderate levels of stress (*M*_*Buddhists*_ = 20.27, *M*_*Christians*_ = 17.63, *M*_*Muslims*_ = 18.35; see Table 1). ANOVA analysis showed a significant difference in the perceived stress scores between the three groups [*F*(2,601) = 10.40, η^2^ = 0.03, *p* < 0.001], with the Buddhist group scored significantly higher on perceived stress than both Christian (*p* < 0.001) and Muslim groups (*p* = 0.05; see Table 2). However, the difference became non-significant after controlling for age and education in ANCOVA, *F*(2,598) = 2.60, η^2^ = 0.01, *p* = 0.076.

**TABLE 2:** *Post hoc* comparison on perceived stress, illness perception, and religious expression by religious groups.

	**Religious groups**		
	**Buddhists vs. Christians**	**Buddhists vs. Muslims**	**Christians vs. Muslims**
**Perceived stress**			
Mean difference (Standard error)	2.64 (0.59)***	1.92 (0.80)*	–0.72 (0.79)
Bonferroni Adj. 98% CI	1.05, 4.24	–0.27, 4.11	–2.88, 1.43
**Illness perceptions**			
*Consequences domain (Item 1)* ^†^			
Mean difference (Standard error)	0.20 (0.21)	0.78 (0.27)*	0.58 (0.27)
Bonferroni Adj. 98% CI	–0.38, 0.77	0.04, 1.52	–0.16, 1.33
*Comprehensibility domain (Item 5)* ^†^			
Mean difference (Standard error)	0.16 (0.15)	–0.63 (0.19)**	–0.78 (0.19)***
Bonferroni Adj. 98% CI	–0.24, 0.55	–1.13, –0.12	–1.29, –0.28
*Emotions domain (Item 6)* ^†^			
Mean difference (Standard error)	0.44 (0.24)	–0.21 (0.31)	–0.65 (0.31)
Bonferroni Adj. 98% CI	–0.21, 1.10	–1.05, 0.64	–1.50, 0.19
**Religious expression**			
*External religious expression* ^†^			
Mean difference (Standard error)	–1.71 (0.09)***	–1.20 (0.11)***	0.51 (0.11)***
Bonferroni Adj. 98% CI	–1.95, –1.47	–1.51, –0.89	0.20, 0.82
*Internal religious expression* ^†^			
Mean difference (Standard error)	–1.38 (0.08)***	–1.40 (0.10)***	–0.03 (0.10)
Bonferroni Adj. 98% CI	–1.59, –1.16	–1.68, –1.13	–0.30, 0.25

^†^Adjusted for covariates (age and education level).

*p < 0.05; **p < 0.01; ***p < 0.001.

Hypothesis 1b: There would be differences in the illness perception domains of the three religious groups without a specific direction.

There were significant differences in three BIPQ illness perceptions between the three religious groups: consequences [*F*(2,601) = 4.94, η^2^ = 0.02, *p* = 0.007], comprehensibility [*F*(2,601) = 7.29, η^2^ = 0.02, *p* = 0.001], and emotions [*F*(2,601) = 6.84, η^2^ = 0.02, *p* = 0.001]. However, after controlling for age and education variables, the emotions domain became non-significant [*F*(2,598) = 2.85, η^2^ = 0.01, *p* = 0.059]. Both the consequences domain [*F*(2,598) = 4.09, η^2^ = 0.01, *p* = 0.017] and the comprehensibility domain [*F*(2,598) = 9.00, η^2^ = 0.03, *p* < 0.001] remained statistically different across three groups.

*Post hoc* analyses showed that the Buddhist group scored significantly higher on the BIPQ consequences domain than the Muslim group (*p* < 0.05). In contrast, the Muslim group scored significantly higher on the BIPQ comprehensibility domain in comparison to Buddhist (*p* < 0.01) and Christian groups (*p* < 0.001) (Table 2). No significant mean differences were found on the other BIPQ domains.

Hypothesis 1c: There would be differences in the religious expression domains of the three religious groups without a specific direction.

There were significant differences in the religious expression between the religious groups [*F*(2,601)_external_ = 243.17, *p* < 0.001, with a large effect size, η^2^ = 0.45; *F*(2,601)_internal_ = 228.74, *p* < 0.001, with a large effect size, η^2^ = 0.43]. These results remained significant after controlling for age and education background with ANCOVA, *F*(2,598)_external_ = 194.83, *p* < 0.001, with a large effect size, η^2^ = 0.39; [*F*(2,598)_internal_ = 183.24, *p* < 0.001, with a large effect size, η^2^ = 0.38].

*Post hoc* analysis showed that Christian and Muslim groups scored significantly higher in ER and IR expression than the Buddhist group (*p* < 0.001). The Christian group also had significantly higher ER expression than the Muslim group (*p* < 0.001; Table 2). The ER of all three religious groups were significantly lower than their IR expression (*p* < 0.001).

Hypothesis 2a: There would be a significant negative relationship between religious expression and perceived stress.

Pearson correlation analyses revealed that, regardless of religious groups, perceived stress was negatively correlated with both ER and IR expression (*p* < 0.001; Table 3). The results remained statistically significant after fractioning out the effects of all eight BIPQ domains (see Supplementary Table 1), thus supporting Hypothesis 2a. It was further confirmed that both ER and IR were significant predictors for perceived stress in the moderation models (*b_*ER*_* = − 3.76, *p* = 0.005; *b*_*IR*_ = − 4.24, *p* = 0.004; see Table 4).

**TABLE 3:** Correlation matrix of perceived stress, religious expression, and illness perception.

**Variables**	**1**	**2**	**3**	**4**	**5**	**6**	**7**	**8**	**9**	**10**	**11**
1. Perceived stress total score	–										
2. External religious expression	−0.18***	–									
3. Internal religious expression	−0.19***	0.78***	–								
4. (*Consequences*) How much does COVID-19 affect your life?	0.29***	−0.08*	–0.08	–							
5. (*Timeline*) How long do you think the COVID-19 pandemic will continue?	0.17***	−0.12**	−0.11**	0.15***	–						
6. (*Personal control*) How much control do you feel you have over the COVID-19 pandemic?	−0.18***	0.05	0.10**	–0.07	0.02	–					
7. (*Concern*) How concerned are you about the COVID-19 pandemic?	0.05	0.03	0.10**	0.28***	0.09*	0.15***	–				
8. (*Comprehensibility*) How well do you feel you understand the COVID-19 pandemic?	−0.16***	0.08*	0.12**	0.01	0.06	0.25***	0.26***	-			
9. (*Emotions*) How much does the COVID-19 pandemic affect you emotionally (e.g., does it make you angry, scared, upset, or depressed)?	0.50***	−0.14***	−0.11**	0.43***	0.16***	–0.04	0.26***	0.02	–		
10. (*Severity*) How severe do you think of COVID-19 as a disease?	0.09*	0.04	0.08*	0.13***	0.17***	0.03	0.38***	0.22***	0.18***	–	
11. (*Likelihood of contracting*) How likely do you think you would contract the COVID-19?	0.20***	–0.06	–0.02	0.16***	0.16***	−0.12**	0.05	–0.04	0.19***	0.02	–

*p < 0.05; **p < 0.01; ***p ≤ 0.001.

**TABLE 4:** Regression coefficients for the relationship between illness perception and perceived stress moderated by religious expressions.

**Variables**	***b*[95% CI]**	***SE***	***p***	***sr*^2^**	***R*^2^**	**Adj. *R*^2^**	**Δ*R*^2^**	**ΔF**
**Model A**								
**Step 1**					0.113	0.110	0.113	38.44***
Age	–0.19 [–0.23, –0.15]	0.02	<0.001	0.115				
Education	–0.56 [–1.44, 0.32]	0.45	0.210	0.002				
**Step 2^a^**					0.354	0.342	0.240	24.47***
BIPQ personal control	–0.25 [–0.44, –0.07]	0.10	0.008	0.008				
BIPQ comprehensibility	–0.41 [–0.71, –0.11]	0.15	0.008	0.008				
BIPQ emotions	1.07 [0.87, 1.26]	0.10	<0.001	0.126				
**Step 3^yb^**					0.370	0.350	0.016	1.91
ER	–3.76 [–6.36, –1.16]	1.32	0.005	0.009				
BIPQ personal control	–0.70 [–1.23, –0.16]	0.27	0.011	0.007				
BIPQ emotions	0.88 [0.28, 1.47]	0.30	0.004	0.009				
ERxBIPQ likelihood of contracting	0.18 [0.01, 0.34]	0.08	0.034	0.005				
**Model B**								
**Step 1**					0.113	0.110	0.113	38.44***
Age	–0.19 [–0.23, –0.15]	0.02	<0.001	0.115				
Education	–0.56 [–1.44, 0.32]	0.45	0.210	0.002				
**Step 2^c^**					0.355	0.343	0.242	24.64***
BIPQ personal control	–0.25 [–0.44, –0.07]	0.10	0.008	0.008				
BIPQ comprehensibility	–0.41 [–0.71, –0.11]	0.15	0.008	0.008				
BIPQ emotions	1.07 [0.87, 1.26]	0.10	<0.001	0.126				
**Step 3^d^**					0.370	0.350	0.015	1.78
IR	–4.24 [–7.09, –1.39]	1.45	0.004	0.009				
BIPQ personal control	–1.03 [–1.74, –0.33]	0.36	0.004	0.009				
IRxBIPQ personal control	0.18 [0.01, 0.34]	0.08	0.034	0.005				

*CI, confidence interval; SE, standard error.*

*^*a*^Predictors entered to Step 2 of Model A include BIPQ consequences, BIPQ timeline, BIPQ personal control, BIPQ concern, BIPQ comprehensibility, BIPQ emotions, BIPQ severity, BIPQ likelihood of contracting, and ER. Only significant variables of interest were presented in the table.*

*^*b*^Predictors entered to Step 3 of Model A include interaction terms between ER and all eight BIPQ variables. Only significant interactions and variables of interest were presented in the table.*

*^*c*^Predictors entered to Step 2 of Model B include BIPQ consequences, BIPQ timeline, BIPQ personal control, BIPQ concern, BIPQ comprehensibility, BIPQ emotions, BIPQ severity, BIPQ likelihood of contracting, and IR. Only significant variables of interest were presented in the table.*

*^*d*^Predictors entered to Step 3 of Model B include interaction terms between IR and all eight BIPQ variables. Only significant interactions and variables of interest were presented in the table.*

Hypothesis 2b: There would be a significant positive relationship between certain illness perception domains (consequences, timeline, concern, emotions, severity, and likelihood of contracting) and a negative relationship between certain illness perception domains (personal control and comprehensibility) with perceived stress.

As hypothesized, significant positive correlations were found between perceived stress and the illness perception domains of consequences, timeline, emotions, severity, and the likelihood of contracting. In addition, significant negative correlations were found between perceived stress and the illness perception domains of personal control and comprehensibility (Table 3). The significance of these correlations remained unchanged after fractioning out the effects of ER and IR expression (See Supplementary Table 2). The only non-significant relationship was between the concern domain and perceived stress.

Another additional multiple regression analysis with perceived stress as the outcome and all eight illness perception domains as the predictors was conducted to further assess the direction of the relationship. The results showed that the illness perception domains of consequences (*b* = 0.25, *p* = 0.033), timeline (*b* = 0.33, *p* = 0.021), emotions (*b* = 1.16, *p* < 0.001), and likelihood of Contracting (*b* = 0.22, *p* = 0.039) were significant positive predictors of perceived stress; whilst personal control (*b* = −0.28, *p* = 0.004) and comprehensibility (*b* = −0.59, *p* < 0.001) were significant negative predictors of perceived stress (see Supplementary Table 3). When age and education was entered as covariates, only the three illness perception domains (personal control, comprehensibility, and emotions) remained as significant predictors of perceived stress with emotion accounting for the largest variances (*b*_Personal Control_ = −0.25, *p* = 0.008; *b*_Comprehensibility_ = −0.41, *p* = 0.008; *b*_Emotions_ = 1.07, *p* < 0.001; see Table 4).

Hypothesis 2c: The relationship between the illness perception domains and perceived stress would be moderated by religious expression, with ER enhancing stress, and IR reducing stress.

Two moderated multiple regressions were conducted, with perceived stress as the outcome variable, age, and education as the covariates, all eight illness perception domains (BIPQ) as the predictors, and ER and IR as moderators (see Models A and B in Table 4). Covariates were entered in the first block, followed by the predictors, and the interaction terms were entered in the third block.

**FIGURE 2:**
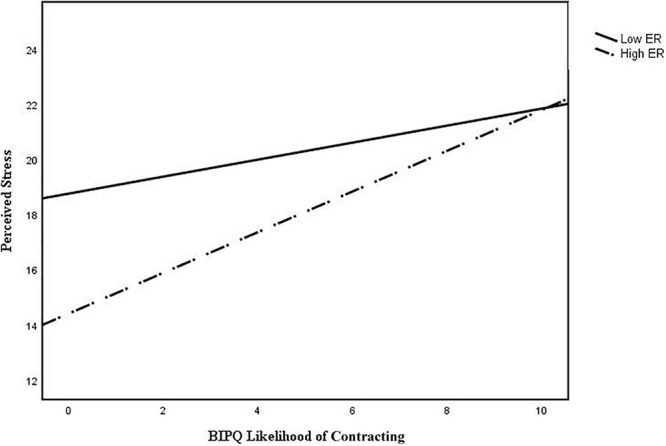
Interaction plot of BIPQ likelihood of contracting and perceived stress with external religious (ER) expression as a moderator.

As hypothesized, the interaction between ER and likelihood of contracting was significant (*b*_ERxBIPQ8_ = 0.18, *p* = 0.034). An examination of the interaction plot found that ER enhanced the positive relationship between likelihood of contracting and perceived stress (see Figure 2). *Post hoc* simple slopes analysis showed that the gradient slope for 1 SD below the mean ER score was −0.047, *p* = 0.0737, and the gradient slope for 1 SD above the mean ER score was 0.398, *p* = 0.003.

**FIGURE 3:**
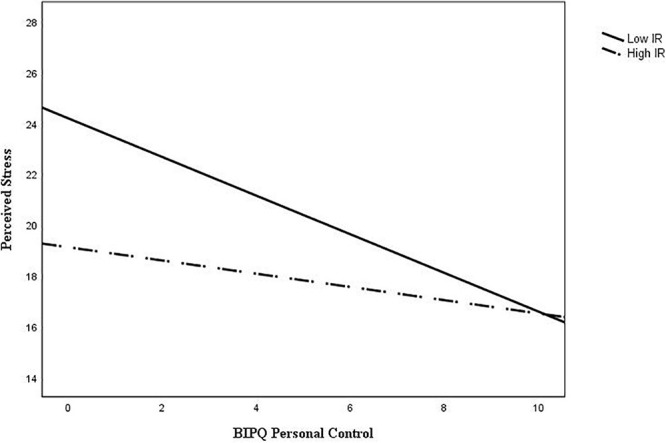
Interaction plot of BIPQ personal control and perceived stress with internal religious (IR) expression as a moderator.

As hypothesized, the interaction between IR and Personal Control was also found to be significant (*b*_BIPQ3xIR_ = 0.19, *p* = 0.026). An examination of the interaction plot found that IR weakened the negative relationship between personal control and perceived stress (Figure 3). *Post hoc* simple slopes analysis showed that the gradient slope for 1 SD below the mean IR score was −0.485, *p* < 0.001, and the gradient slope for 1 SD above the mean IR score was −0.051, *p* = 0.432.

### Qualitative Analysis: Illness Causation

A total of 1,847 written responses (of 608 participants) were identified and extracted from the BIPQ causal representation open-ended question pertaining to the perceived factors causing the Covid-19 pandemic. Inductive-deductive thematic analysis yielded seven major themes (by ranking)—consequences of human behaviors, consequences of human attitudes, sociopolitical reasons, social factors, medical explanations, ecological explanations, and religious-spiritual explanations. A derived codebook with themes, subthemes, and illustrative quotes (examples) was shown in Table 5. Following are the illustration of each major theme (by percentage ranking; see Supplementary Table 4):

**TABLE 5:** Themes, subthemes, and examples of pandemic causal representation (*N* = 608).

**Themes**	**Subthemes**	**Examples**
**Consequences of human behavior**		
	Poor public health behavior	“Lack of hygiene” “No social distancing” “Not washing hands enough”
	Unusual eating choices and behavior	“Consuming wildlife” “Consumption of exotic meat” “Poor food choices”
	Public fail to follow government protocols	“Citizen not following MCO rules” “Human disobedience” “Public did not follow official rules”
	Environment disaster caused by humans	“Humans looting Earth’s resources without limitations” “Human abuse of the environment” “Human encroachment into wildlife”
	Uncooperative community	“Public less cooperative” “Humans not cooperating with the government”
	High risk individuals not following protocols	“Sick individuals not seeking medical help” “Fear of discrimination in disease disclosure leading to concealment of travel history” “Unrestricted movements of people with symptoms”
	Unhealthy lifestyle	“Human lifestyle” “Living environment” “Unhealthy lifestyle”
	Reckless behavior	“Irresponsible behavior” “Lack of self-discipline”
	Fake news	“Spread of false news” “Falsified information”
**Consequences of human attitudes**		
	Lack of awareness and education	“Awareness in society not enough” “Lack of awareness and education regarding the virus and its severity” “The knowledge about COVID-19”
	Human flawed characters	“Arrogance” “Selfishness of humans” “Stubbornness”
	Human ignorance	“Ignorant individuals who refused to be tested” “Human negligence”
	Underestimated the severity of virus	“People think it is not a concern” “People underestimating the virus” “Not serious in preventing the outbreak in the beginning”
	Public mindset	“Lack of social responsibility” “Irresponsible attitude”
	Human attitudes	“Too complacent” “Attitude towards the pandemic”
	Public emotional reaction	“Fear in society” “Lack of proper planning, causing panic”
**Socio-political reasons**		
	Ineffective government	“Government fail to take action at the early stage” “Government’s effectiveness in decision and action” “Lack of border control at the right time between countries”
	Man-made disaster	“Bio weapon” “It is also a conspiracy by pharmaceutical and parties with vested interests to gain control” “Leak in scientific experiment”
	Poor medical resources	“Insufficient medical equipment for front line health personnel” “Unprepared-ness for pandemics (hospitals etc.)” “Lack of funding in preventive measures and healthcare in general”
	Poor preventive measures	“Ineffective precaution measures” “Did not have proper preventive measures” “Lack of immediate preventive measures”
	Caused by China	“China’s cover up” “Poor management and late information from China” “Wuhan wetmarket”
	Economical factor	“Over development” “Most of country more concern the economic factor rather the health and safety of the people” “Economic factors”
	International politics	“Egoism between politicians” “A well implemented containment policy and mitigation measures by solidarity of national and global level plays imperative role in handling this pandemic” “Lack of warning and reminder from WHO at the early stage of epidemic”
	Caused by United States	“It is a political ploy by US to sabotage China’s progress economically and politically” “Some people say this virus was originally from United States, brought to China”
**Social factors**		
	Social gathering	“Mass gathering” “Freely gathering in virus risk areas” “Social activities”
	Human interaction	“Close contact between humans” “Exposure to many people” “Overcrowding”
	Human mobility	“Globalization of the world” “People going from places to places” “Increasing ease and extent of global travel in recent times that facilitates the worldwide spread of the outbreak”
	Human existence	“Human factor” “Human activities” “Human behavior”
	Space sharing	“Going to public places often” “Going to crowded places with no air circulation” “Crowded population in housing area”
	Religious factor	“Thinking religion will save them” “Religious gathering”
	Cultural factor	“Culture” “Different ways of dealing with disease (e.g., there’s a difference in way of approach in cultures (e.g., western and Asian)”
**Medical explanation**		
	Poor immune system	“Low immune system” “Preexisting diseases” “Health conditions”
	Virus transmission	“Rapid spread of virus” “Highly contagious virus” “Present of virus load”
	Infections	“Contracted through contaminated air droplets” “Unexpectedly infected” “Transmission from human to human without symptoms”
	Bio-mutation	“Genetic mutation” “Virus mutation” “Newness of the virus”
	Contact with COVID-19 positive cases	“Close contact with infected people” “Interacting with people who is displaying symptoms”
	Physical contact	“Physical contact with humans” “Hand shaking” “Spread through touch”
	Hard to detect	“Existence of asymptomatic patients” “Difficulty in detecting carriers” “COVID-19’s low fatality rate and asymptomatic disease state, which translate into a sizeable number of asymptomatic carriers of SARS-CoV-2, consequently increasing the chance of spreading the virus”
	Physical symptoms	“Cough” “Breathing difficulties”
	High risk and severity rate	“High contagious rates” “High mortality rates”
	Microbiology transmission	“Bacteria (like black plague)” “Airborne bacteria transmission in a closed space”
**Ecological explanation**		
	Environment problem	“Global warming” “Polluted environment”
	Natural process	“Natural occurrence” “What we contribute to the world it comes back to us” “Seasonal bound to happen”
	Animal contact	“Spread from animal” “Bat or any other animals”
	Population problem	“Overpopulation of humans” “Population density”
	Natural disaster	“Natural disasters”
**Religious-spiritual explanation**		
	Karma/Sin	“Karma” “Sin of men”
	Will of God	“God’s will” “God wants all humans to return back to Him, love Him, worship Him” “Power of Allah”
	Punishment from God	“God’s punishment” “God’s judgment and wrath” “God’s power in reprimanding His slave”
	Fatalism	“End of the world, already predicted” “Destiny”
	Testing from God	“Lesson to learn from God”
	Personal faith	“Ignoring God” “Poor spirituality”

#### Consequences of Human Behaviors

Consequences of human behaviors were defined as any external human behaviors that violate the social norms of individuals, groups, or communities (inclusive of virtual communities) that led to the pandemic. For example, the majority of the responses indicated that poor public health behaviors (15.86%), such as “no social distancing,” “bad hygiene habits,” “did not wear a mask,” and “poor food hygiene,” were the reasons behind the pandemic. Another subtheme was unusual eating choices and behaviors (4.89%), where “wildlife consumption” and “eating exotic animals” were deemed the reasons behind the pandemic. The third subtheme was the public’s failure to follow government protocols (3.91%), where “citizen not following MCO rules” and “human disobedience” were cited as examples. Other subthemes included environmental disasters caused by humans (1.3%), uncooperative community (1.2%), high-risk individuals not following protocols (1.03%), unhealthy lifestyle (0.92%), reckless behavior (0.65%), and fake news (0.33%).

#### Consequences of Human Attitudes

Consequences of human attitudes were defined as any human factors caused by internal processes, including emotion, rationality, human characters, and individual and public mindsets that caused the pandemic. A subtheme of consequences of human attitudes was a lack of awareness and education (8.37%), such as “lack of awareness and education regarding the virus and its severity,” which contributed to the spread of the pandemic. Another subtheme was human’s flawed characters (5.81%), where it was mentioned that the pandemic was caused by the moral implications of human characters or virtues, such as “greed,” “dishonesty,” “stubbornness,” “arrogance,” and “carelessness.” Human ignorance (4.78%) was also a subtheme of consequences of human attitudes, where responses showed that “general public ignorance” and “public indifference” caused the pandemic. Similarly, responses also revealed that underestimating the severity of the virus (2.61%), public mindset (1.25%), human attitudes (1.25%), and public emotional reaction (0.65%) were deemed as causes behind the pandemic.

#### Sociopolitical Reasons

Sociopolitical reasons included local and foreign politics, government policies and resources, political agendas, and conspiracy theories contributing to the spread of the pandemic. One subtheme of sociopolitical reasons was ineffective government (5.59%). Examples of ineffective government included the government’s “slow response to the pandemic,” “selfishness of government,” “lack of reinforcement,” “corrupted politics,” and “change of government” during this critical period. Responses also reflected the pandemic as a human-origin disaster (1.85%), caused by “bioweapon,” “methodology of food production,” and “intentional actions to release the virus for gains” of vested parties. Another subtheme of sociopolitical reasons included poor medical resources (1.74%), where the “lack of medical resources and PPE” and “insufficient medical funding” perpetuated the pandemic. Other subthemes include poor preventive measures (1.2%), caused by China (1.09%), economic factors (1.09%), international politics (0.87%), and caused by United States (0.16%).

#### Social Factors

Social factors reflected any external factors causing the pandemic due to social environments and group interactions, such as religious affiliation, cultural norm, and human mobility. Two main subthemes of social factors were social gathering (3.75%) and human interaction (3.26%). Examples of social gathering and human interaction include “mass gathering” and “close contact between humans,” respectively. Human mobility (1.96%) was also attributed as one of the factors behind the pandemic, such as the ease of “global travel” and “people going from place to place.” Responses also indicated that human existence (1.52%), space sharing (0.98%), religious factors (0.71%), and cultural factors (0.22%) were the causes behind this pandemic.

#### Medical Explanation

Medical explanation referred to biological perspectives on the cause of the pandemic based on the scientific facts and terminologies. Responses attributed poor immune systems (3.1%), such as “low immune system” and “preexisting conditions,” as one of the causes behind the pandemic. Virus transmission (2.39%) was another subtheme of medical explanations. Examples of virus transmission included “a rapid spread of the virus” and “the presence of virus load.” Another subtheme of medical explanation was infections (1.63%), defined as the method or pathway of infection, including “infected by humans,” “cross-contamination,” “zoonosis,” and “contracted through contaminated air droplets.” Responses also indicated that the pandemic was caused by bio-mutation (1.36%), such as “virus mutation” and “genetic mutation.” Other subthemes within the medical explanation theme were—contact with Covid-19 positive cases (0.87%), physical contact (0.65%), hard to detect (0.49%), physical symptoms (0.43%), high-risk and severity rates (0.22%), and microbiology transmission (0.11%).

#### Ecological Explanation

The ecological explanation was defined as attributing the cause of pandemic to natural courses of development, such as population issues, environmental pollution, and natural disaster. Subthemes of ecological explanation included environment problem (2.23%), natural process (0.98%), animal contact (0.76%), population problem (0.65%), and natural disaster (0.16%). Examples of these subthemes were “polluted environment,” “natural selection,” “virus transmitted through animals/bats,” “overpopulation,” and “nature’s disease,” respectively.

#### Religious-Spiritual Explanation

Some participants adopted religious and spiritual perspectives rooted in doctrines of religious teachings as causal explanations of the pandemic. A subtheme of religious-spiritual explanation was karma/sin (0.65%), where responses indicated that the pandemic is the “karma” caused by the “sin of men.” Some also referred to the pandemic was a will of God (0.6%), where it was “God’s act” and “God’s testing” for humans. Another subtheme of religious-spiritual explanation was that the pandemic was a punishment from God (0.54%; “God’s judgment and wrath”). Other subthemes included fatalism (0.27%; “apocalypse”), testing from God (0.16%; “lesson to learn from God”), and personal faith (0.16%; “poor spirituality”).

In summary, all religious groups endorsed “human behavior and characters” as major reasons of pandemic, according to their cultural beliefs. Medical and religious-spiritual explanations were relatively minor compared to the social-political factors.

## Discussion

### Religion as a Double-Edged Sword During the Covid-19 Pandemic

Firstly, this study revealed that a higher level of religiosity was associated with lower stress levels during the lockdown regardless of the form of religious practice. The results are consistent with the past empirical findings where higher intrinsic religiosity and spirituality were associated with better health, subjective well-being, reduced depressive and posttraumatic stress symptoms, and reduced stress (Arévalo et al., 2008; Power and McKinney, 2013; Chen and VanderWeele, 2018; You and Lim, 2018; Villani et al., 2019). A recent study on the religious communities of the United Arab Emirates affected by Covid-19 suggested that positive religious coping was associated with the reduced risk of depression among Muslims during the pandemic (Thomas and Barbato, 2020).

However, many past studies used the Religious Coping Scale (RCOPE; Pargament et al., 2011) to measure religious coping, which examines mainly IR expression, and omitted external and communal religious coping (e.g., mass religious gathering and incense burning), although findings pointed out that ER engagement promoted stress resilience and provided support by uniting its followers through religious congregations (Graham and Haidt, 2010; Brewer-Smyth and Koenig, 2014; Mojahed, 2014). Similarly, Tartaro et al. (2005) found that composite religiosity (a combination of religious involvement, engagement, church attendance, religious coping, private religious practices, and overall self-ratings of religiosity) were significantly associated with lower cortisol levels. Our study suggested that both internal religiosity as well as ER expression (e.g., organized religious behaviors) were associated with lower stress levels experienced by the believers during the pandemic lockdown.

The mechanism of ER expression as a potential protective factor may be further understood *via* the Peircean triadic sign theory. Through Peircean semiotic analysis (Atkin, 2013; Ting et al., 2020), religious participation can be seen as a form of “index” in an intact sign of religion. Besides relying on the “symbol” (religious teaching) and “icon” (religious leader), a triadic sign system needs an “index” (believers’ participation in rituals) to be complete. When a cultural sign system is intact, emotional transformation and self-transcendence are achieved (Sundararajan, 2011). This triadic sign is displayed in the “interdependent” self-construal embedded in many collective societies (Markus and Kitayama, 1991), despite modernization and globalization in the 21st century. During the Covid-19 pandemic time, what overrides “scientific principles” of illness perception is this need to restore “cultural sign system” in many Asian ethno-religious groups, for example, the “cow dung or cow urine treatment” practiced by the public in India (Ghangar, 2021). As mass religious gatherings are against the scientific rules of “social distancing,” many religions very quickly converted their weekly gatherings into a virtual realm through online platforms. Therefore, our results showed that higher internal religiosity could lessen the stress associated with the loss of personal control. However, not all religious ceremonies are “easily convertible” to online rituals, hence creating further stressors for some Asian devotees to seek other forms of ER practices that escalate the risk of the Covid-19 outbreak. For instance, a recent study found that the Covid-19 pandemic outbreak had associations with religious tourism and mass religious gatherings (Mubarak and Zin, 2020). On the other hand, our results also showed that high external religiosity increases the stress associated with a higher perceived likelihood of Covid-19 infection.

Hence, religious resources and community seem to be a double-edged sword providing believers a way to regulate pandemic stress while, on the other hand, increasing the stress of being infected by the virus due to physical gatherings. The protection of the public and the employment of religious coping create a social dilemma for certain religious groups and the government. For example, despite the continuous increase of daily Covid-19 cases in May, 2 months into the implementation of MCO, the Malaysian government allowed the reopening of houses-of-worship in Covid-free zones (New Straits Times, 2020). According to the religious affairs minister of Malaysia, congregational prayers were permitted as “even though worship in Islam is not confined only to [houses-of-worship], it has a profound effect on the spiritual development of Muslims” (The Straits Times, 2020). Similarly, Catholic churches in the Archdiocese of Kuala Lumpur planned to resume its masses and sacrament celebrations following strict government guidelines (Ting and Perimbanayagam, 2020). However, other religious leaders have opted to delay the reopening of their religious sites in prioritizing safety of the congregations. The Council of Churches of Malaysia announced that “in keeping with our spiritual obligations and social responsibility, we shall continue to pray and worship at our homes, and also offer online streaming of our worship services” (Radhi, 2020, para. 7). This sentiment was also echoed by the Malaysian Buddhist Association and Malaysian Hindu Sangam (Ting and Perimbanayagam, 2020). It is anticipated that the changes in religious ecology would continue to co-evolve with the forms of religious expression available to the community.

### Religious Ecologies Inform Illness Representations

Secondly, as predicted, different religious groups were confirmed to vary in their illness perception. By a narrow margin, the Muslim group reported the highest confidence in their knowledge (perceived comprehensibility) of Covid-19 compared to two other groups. This is most likely due to the available Covid-19 information channeled mainly in the national language—Malay, a dominant language for the Muslim community; whereas the majority of our participants from Buddhist and Christian groups were from the minority groups—Chinese and Indians, who therefore might have less direct access to pandemic-related information due to the lack of a variety of language mediums in news releases (Wang, 2016). On the other hand, albeit with a slight difference, the Buddhist group was found to be most “affected” due to the consequence of Covid-19 pandemic, comparing to their counterparts. In Malaysia, since most self-identified Buddhists practice a mix of folk religions from Chinese Taoism and Maharaja Buddhism tradition, which focuses mainly on ritual practice and ancestor worship (Ahmad, 2007; Tang, 2015; Samuels, 2017; Ting et al., 2020), the physical lockdown of religious venues could have affected them the most.

The differences of pandemic cognitive representation across the three groups are also in synchrony with their causal attributions of the pandemic. Though all of them attributed the pandemic as human doing (behaviors) and being (nature), the Christian group seems to have a higher ranking on medical explanation and sociopolitical reasons than the other two groups. When examining closer into the subthemes that fall under these two categories, many of them are conceptual and abstract in nature, such as systemic problems (e.g., ineffective government, man-made disaster, and international politics) and scientific analysis (e.g., virus transmission, poor immune system, bio-mutation, microbiology, etc.). As previously studied, Christianity stems from a weak-ties society (Ting and Sundararajan, 2018; Schulz et al., 2019) that privileges abstract and analytic cognitive styles. These variations in causal attribution are worthy of future investigation through the measurement of specific cognitive styles across different religious groups.

### Implications on Healthcare and Mental Health Practices

Though our study did not establish a causal relationship between religiosity and well-being, the negative association between religious expression and stress could have several implications for the healthcare practitioners. In the past literature, religious involvement has been proven to be an effective tool in shaping health behavior regulation, due to its positive impact on self-control and self-regulation (McCullough and Willoughby, 2009; Aldwin et al., 2014). Therefore, the religious community could be further encouraged to assist in the prevention of pandemic, such as by being proactive in Covid-19 screening tests, adhering to health behaviors and safety protocol, promoting vaccination, de-stigmatizing the diagnosis of Covid-19, volunteering for community outreach, acknowledging human errors and self-centered tendencies, and building global solidarity in the midst of a shared fate. To reach out to the religious communities, it is important to engage religious leaders in the planning of health promotion programs and the training of healthcare personnel in enquiring for religious and spiritual beliefs of patients during clinical assessment (Tan et al., 2021). Koenig (2020) also advocated for religious faith as an essential resource for health and psychological well-being during this critical period. Learning about the unique worldviews and perceptions toward pandemic in each religious community also enables public health policies in working collaboratively with religious leaders to promote safety behaviors and in delineating cultural-sensitive interventions. For example, in rural Muslim-Malay communities, some villagers have utilized concrete spiritual symbols (e.g., dressing up as Covid-19 ghosts) to reinforce the “stay-home” behaviors among villagers, which was deemed effective by the locals (The Star, 2020b).

The significant relationship between emotional response, personal control, Covid-19 comprehension, and stress, also suggests that mental health practitioners may help the public to reduce stress by boosting emotional stability, personal control, and increasing Covid-19 knowledge. Should a causal link be established in the future, public health propaganda could also attempt to diffuse the negative emotion triggered by Covid-19, rather than provoke fear or shame through exaggerated propaganda and idiosyncratic cases. Cultivating a social atmosphere of optimism and acceptance is a potential future direction for policymakers.

### Limitations and Future Directions

Though with meaningful findings, this study is not without limitations. The current sample consists mainly of the Chinese ethnic group, which is not representative of the Malaysian demographic. Due to the snowballing sampling method, there is also a significant difference in age, education, and residences across the three religious groups. The Muslim group also consisted of a much smaller sample size than the other two religious groups, which may confound statistical analyses. Additionally, as the study was conducted anonymously *via* emails and social networking sites, there is a possibility of nested data, with participants coming from the same family and community, and hence potentially violating the assumption of statistical independence of data. A future study could address these limitations by using stratified sampling to recruit a more diverse demographic sample and control for socio-economic status variables (e.g., income level and employment status). The inclusion of more Muslims and Indian participants would improve the generalizability of this study finding to overall Malaysian populations. Nevertheless, one unique feature of Malaysian religious community is the intersectionality of religion and ethnicity (for example, most of the Chinese are Buddhists, and all Malays are defaulted Muslims). The generalizability and interpretation of the results should be mindful of such intersections.

In terms of methodology, future research could further diversify the measurement of religiosity to include innovative worship participation (e.g., online services) to reflect the new normal of religious participation during the pandemic. The definition of ER vs. IR expressions in this study was mainly based on the feasibility of these expressions during the pandemic lockdown. *Post hoc* confirmatory factor analysis (CFI) showed borderline acceptability of this two-factor model (CFI = 0.92). A more sophisticated measurement may be developed to tap into these two dimensions of religiosity in future, by separating religious activities and beliefs. In the current study, Buddhist group scored significantly lower than the other two groups in religious expression. This could be due to the diffused boundaries of Buddhism and folk religion practiced in Malaysia, which is difficult to capture through the ADUREL items. In future, communal religious or spiritual rituals may be measured by frequencies of religious practices specific to each religious group (such as practicing *tai-ji* for Buddhist-Taoist group), rather than the degree of agreement.

Furthermore, there were some concerns regarding construct validity of BIPQ as the use of single-time subscales poses a risk of random measurement errors and involve higher ambiguity when interpreting the meaning of an item (Hoeppner et al., 2011). *Post hoc* analyses of exploratory factor analysis also indicated a two-factor model with low factor loading that only accounts for 47% of variance. Extended research was conducted using more comprehensive scales such as IPQ-Revised (Moss-Morris et al., 2002), or other validated scales that could be tested for measurement invariance.

In our study, the multi-step regression analysis also revealed that age accounted for the most variance in perceived stress during lockdown. This concurs with the previous finding in Spain that younger populations perceiving more Covid-19 pandemic stress than the overall populations (Ozamix-Etxebarria et al., 2020). This incidental finding about age being the potential protective factor of stress worth’s further exploration in future study. Future studies could also expand the framework of religious impact by including religious coping variables and health behavior or health outcomes across different religious groups. The inclusion of a comparison group of atheists or non-believers would help determine whether religious identification truly makes a difference in pandemic stress regulation. Lastly, though there are several existing multination psychological studies on issues relevant to the Covid-19 pandemic, it will be informative to explore how religion in those countries plays a role in health and mental health system promotion.

## Conclusion

As various religious groups struggle to make the meaning of this crisis through their indigenous ways of knowing, this study addressed the impact of ecological system (culture) toward pandemic responses with understudied religious groups such as Muslims and Buddhists, who make up a majority of the global population. This study found that both external and internal forms of religiosity are associated with lower stress levels; religiosity could also moderate the stress stemming from various illness perceptions toward Covid-19. Globally, many religious communities remain highly active during the pandemic to provide solace and peace in times of uncertainty. However, parallel concern toward religious gatherings exists due to the implied risk of virus transmission. The tension between “scientific” and “religious” traditions needs to be reconciled for devotees to adhere to public health policies of social distancing, and for the policymakers to respect and understand the value of religious ceremonies. This study highlighted the importance of the cultural psychology discipline in informing pandemic prevention policies and having culturally sensitive mental health and public health deliveries in this critical time. As Malaysia is a multiethnic and multireligious country, these results may have implications for other countries with similar religious compositions and heterogeneity in their populations.

## Data Availability Statement

The unidentified raw data supporting the conclusions of this article will be made available by the authors, for verification and auditing purpose upon requests.

## Ethics Statement

The studies involving human participants were reviewed and approved by the Monash University Ethics Review Board. Implied consent from the participants was required for this study in accordance with the national legislation and the institutional requirements.

## Author Contributions

RT contributed to the concept, method, framework, and funding of the study and revised the manuscript. YY-AY contributed to literature review, data collection and analysis of both qualitative and quantitative data, as well as drafting Sections “Methodology” and “Results” of the manuscript. MM-T contributed to data collection and analysis of quantitative data, as well as reviewing the manuscript. CK-Y contributed to the concept of the research paradigm, literature review, and data collection of the project. All the authors participated in the manuscript revision and provided inputs on the final draft.

## Conflict of Interest

The authors declare that the research was conducted in the absence of any commercial or financial relationships that could be construed as a potential conflict of interest.

## Publisher’s Note

All claims expressed in this article are solely those of the authors and do not necessarily represent those of their affiliated organizations, or those of the publisher, the editors and the reviewers. Any product that may be evaluated in this article, or claim that may be made by its manufacturer, is not guaranteed or endorsed by the publisher.

## References

[B1] Abdullah M. (2020). *Staying on Campus During MCO a Strain on Students.* Malaysia: New Straits Times.

[B2] Ahmad Z. (2007). Multiculturalism and religio-ethnic plurality. *Cult. Relig.* 8 139–153. 10.1080/14755610701424008

[B3] Ahmadi F. Mohamed Hussin N. A. Mohammad M. T. (2019). Religion, culture and meaning-making coping: a study among cancer patients in Malaysia. *JORH* 58 1909–1924. 10.1007/s10943-018-0636-9 29948793 PMC6842329

[B4] Aldwin C. M. Park C. L. Jeong Y. J. Nath R. (2014). Differing pathways between religiousness, spirituality and health: a self-regulation perspective. *Psychol. Relig. Spiritual* 6 9–21. 10.1037/a0034416

[B5] Al-Khayat M. H. (2004). “Health as a human right in Islam,” in *The Right Path to Health: Health Education through Religion*, ed. Al-Khayat M. H. (Cairo: World Health Organization).

[B6] Arévalo S. Prado G. Amaro H. (2008). Spirituality, sense of coherence, and coping responses in women receiving treatment for alcohol and drug addiction. *Eval. Program. Plann.* 31 113–123. 10.1016/j.evalprogplan.2007.05.009 17825910

[B7] Atkin A. (2013). “Peirce’s theory of signs,” in *The Stanford Encyclopedia of Philosophy*, ed. Zalta E. N. (Annapolis, MD: CSLI).

[B8] Attum B. Hafiz S. Malik A. Shamoon Z. (2020). *Cultural Competence in the Care of Muslim Patients and their Families.* Treasure Island, Fl: StatPearls Publishing. 29763108

[B9] Aziz N. A. Othman J. Lugova H. Suleiman A. (2020). Malaysia’s approach in handling COVID-19 onslaught: report on the Movement Control Order (MCO) and targeted screening to reduce community infection rate and impact on public health and economy. *J. Infect. Public Health* 13 1823–1829. 10.1016/j.jiph.2020.08.007 32896496 PMC7456294

[B10] Belzen J. (2010). *Towards Cultural Psychology of Religion.* Netherlands: Springer.

[B11] Braun V. Clarke V. (2006). Using thematic analysis in psychology. *Qual. Res. Psychol.* 3 77–101. 10.1191/1478088706qp063oa 32100154

[B12] Brewer-Smyth K. Koenig H. (2014). Could spirituality and religion promote stress resilience in survivors of childhood trauma? *Issues Ment. Health Nurs.* 35 251–256. 10.3109/01612840.2013.873101 24702209

[B13] Broadbent E. Petrie K. J. Main J. Weinman J. (2006). The brief illness perception questionnaire. *J. Psychosom. Res.* 60 631–637. 10.1016/j.jpsychores.2005.10.020 16731240

[B14] Chakraborty I. Maity P. (2020). COVID-19 outbreak: migration, effects on society, global environment and prevention. *Sci. Total Environ.* 728:138882. 10.1016/j.scitotenv.2020.138882 32335410 PMC7175860

[B15] Channel News Asia (2021). *COVID-19: All Malaysian States Except Sarawak to be Placed Under MCO Starting Friday.* Singapore: Channel News Asia.

[B16] Chen Y. VanderWeele T. (2018). Associations of religious upbringing with subsequent health and well-being from adolescence to young adulthood: an outcome-wide analysis. *Am. J. Epidemiol.* 187 2355–2364. 10.1093/aje/kwy142 30215663 PMC6211237

[B17] Chew B. H. Vos R. C. Heijmans M. Shariff-Ghazali S. Fernandez A. Rutten G. (2017). Validity and reliability of a Malay version of the brief illness perception questionnaire for patients with type 2 diabetes mellitus. *BMC Med. Res. Methodol.* 17:118. 10.1186/s12874-017-0394-5 28774271 PMC5543429

[B18] Chew K. S. Tan T. W. Ooi Y. T. (2011). Influence of Chinese cultural health beliefs among malaysian chinese in a suburban population: a survey. *SMJ* 52 252–256. 21552785

[B19] Chong L. T. Chong M. C. Tang L. Y. Ramoo V. Chui L. P. Hmwe N. T. T. (2019). The relationship between psychological distress and religious practices and coping in Malaysian parents of children with thalassemia. *J. Pediatr. Nurs.* 48 e15–e20. 10.1016/j.pedn.2019.05.016 31213340

[B20] Cohen J. (2013). *Statistical Power Analysis for the Behavioral Sciences.* Cambridge, MA: Academic press.

[B21] Cohen S. Williamson G. (1988). ““Perceived stress in a probability sample of the U.S.”,” in *The Social Psychology of Health: Claremont Symposium on Applied Social Psychology*, eds Spacapam S. Oskamp S. (California: SAGE Publications), 31–67.

[B22] Department of Statistics Malaysia (2011). *Population and Housing Census of Malaysia.* Malaysia: Department of Statistics Malaysia.

[B23] Dorall A. (2020). *Spikes in Suicide Nationwide During MCO.* Available online at: https://www.therakyatpost.com/2020/08/11/spike-in-suicides-nationwide-during-mco/ (accessed August 11, 2020)

[B24] Dueck A. (2020). *Indigenous Psychology of Spirituality: in my Beginning is my End.* Switzerland: Palgrave Macmillan.

[B25] Faul F. Erdfelder E. Buchner A. Lang A.-G. (2009). Statistical power analyses using G^∗^Power 3.1: tests for correlation and regression analyses. *Behav. Res. Methods* 41 1149–1160. 10.3758/brm.41.4.1149 19897823

[B26] Fereday K. Muir-Cochrane E. (2006). Demonstrating rigor using thematic analysis: a hybrid approach of inductive and deductive coding and theme development. *Int. J. Qual. Methods* 5 80–92. 10.1177/160940690600500107

[B27] Ghangar G. M. (2021). *Covid Centre Inside ‘gaushala’ in Gujarat Treats Patients with Drugs from Cow Milk, Urine.* Noida: India Today.

[B28] Graham J. Haidt J. (2010). Beyond beliefs: religions bind individuals into moral communities. *Pers. Soc. Psychol. Rev.* 14 140–150. 10.1177/1088868309353415 20089848

[B29] Granovetter M. (1973). The strength of weak ties. *Am. J. Sociol.* 78 1360–1380. 10.1086/225469

[B30] Guo J. Feng X. Wang X. van IJzendoorn M. (2020). Coping with COVID-19: exposure to COVID-19 and negative impact on livelihood predict elevated mental health problems in Chinese adults. *Int. J. Environ. Res. Public Health* 17:3857. 10.3390/ijerph17113857 32485859 PMC7312167

[B31] Hassan H. (2020). *Coronavirus: Mental Health Issues Rise During Malaysia’s Partial Shutdown.* Singapore: The Strait Times.

[B32] Hill P. C. Edwards E. (2013). “Measurement in the psychology of religiousness and spirituality: existing measures and new frontiers,” in *APA Handbook of Psychology, Religion, and Spirituality: Context, Theory, and Research*, eds Pargament K. I. Exline J. J. Jones J. W. (Washington, DC: American Psychological Association), 51–77. 10.1037/14045-003

[B33] Hill P. C. Pargament K. I. (2003). Advances in the conceptualization and measurement of religion and spirituality: implications for physical and mental health research. *Am. Psychol.* 58 64–74. 10.1037/0003-066X.58.1.64 12674819

[B34] Hoeppner B. B. Kelly J. F. Urbanoski K. A. Slaymaker V. (2011). Comparative utility of a single-item versus multiple-item measure of self-efficacy in predicting relapse among young adults. *J. Subst. Abuse Treat.* 41:305. 10.1016/j.jsat.2011.04.005 21700411 PMC3315352

[B35] Hood R. W. J. Hill P. Spilka B. (2018). *The Psychology of Religion: An Empirical Approach*, 5th Edn. New York, NY: Guilford Press.

[B36] IBM Corp. (2017). *IBM SPSS Statistics for Windows.* Armonk, NY: IBM Corp.

[B37] Khodaveirdyzadeh R. Rahimi R. Rahmani A. Ghahramanian A. Kodayari N. Eivazi J. (2016). Spiritual/religious coping strategies and their relationship with illness adjustment among Iranian breast cancer patients. *Asian Pac. J. Cancer Prev.* 17 4095–4099. 27644667

[B38] Koenig H. Büssing A. (2010). The Duke University Religion Index (DUREL): a five-item measure for use in epidemological studies. *Religions* 1 78–85. 10.3390/rel1010078

[B39] Koenig H. Parkerson G. Meador K. (1997). Religion index for psychiatric research. *Am. J. Psychiatry* 154 885–886. 10.1176/ajp.154.6.885b 9167530

[B40] Koenig H. G. (2020). Maintaining health and well-being by putting faith into action during the COVID-19 pandemic. *JORH* 59 2205–2214. 10.1007/s10943-020-01035-2 32409989 PMC7224109

[B41] Leung D. Lam T. Chan S. (2010). Three versions of perceived stress scale: validation in a sample of Chinese cardiac patients who smoke. *BMC Public Health* 10:513. 10.1186/1471-2458-10-513 20735860 PMC2939644

[B42] Leventhal H. Leventhal E. A. Contrada R. J. (1998). Self-regulation, health, and behavior: a perceptual-cognitive approach. *Psychol. Health* 13 717–733. 10.1080/08870449808407425

[B43] Lippi G. Henry B. Bovo C. Sanchis-Gomar F. (2020). Health risks and potential remedies during prolonged lockdowns for coronavirus disease 2019 (COVID-19). *Diagnosis* 7 85–90. 10.1515/dx-2020-0041 32267243

[B44] Malay Mail (2021). *Covid-19: 85.5pc of Distress Calls Involved Mental Health Issues, Says Health Ministry.* Malaysia: Malay Mail.

[B45] Marin M. Lord C. Andrews J. Juster R. Sindi S. Arsenault-Lapierre G. (2011). Chronic stress, cognitive functioning and mental health. *Neurobiol. Learn. Mem.* 96 583–595. 10.1016/j.nlm.2011.02.016 21376129

[B46] Markus H. R. Kitayama S. (1991). Culture and the self: implications for cognition, emotion, and motivation. *Psychol. Rev.* 98 224–253. 10.1037/0033-295X.98.2.224

[B47] McCullough M. Willoughby B. (2009). Religion, self-regulation, and self-control: associations, explanations, and implications. *Psychol. Bull.* 135 69–93. 10.1037/a0014213 19210054

[B48] Miceli J. Geller D. Tsung A. Hecht C. L. Wang Y. Pathak R. (2019). Illness perceptions and perceived stress in patients with advanced gastrointestinal cancer. *Psycho-Oncol* 28 1513–1519. 10.1002/pon.5108 31090125 PMC6610754

[B49] Milstein G. Palitsky R. Cuevas A. (2019). The religion variable in community health promotion and illness prevention. *J. Prev. Int. Commun.* 48 1–6. 10.1080/10852352.2019.1617519 31402789

[B50] Minhat H. Shahar H. K. (2020). The trajectory of COVID-19 scenario in Malaysia: facing the unprecedented. *Curr. Med. Res.* 36 1309–1311. 10.1080/03007995.2020.1786680 32569488

[B51] Mojahed A. (2014). Religiosity and preventing risky behaviors. *Int. J. High Risk Behav. Addict.* 3:e22844. 10.5812/ijhrba.22844 25593894 PMC4286922

[B52] Montano R. Acebes K. (2020). Covid stress predicts depression, anxiety and stress symptoms of Filipino respondents. *IJRBS* 9 78–103. 10.20525/ijrbs.v9i4.773

[B53] Morse J. (2015). Critical analysis of strategies for determining rigor in qualitative inquiry. *Qual. Health Res.* 25 1212–1222. 10.1177/1049732315588501 26184336

[B54] Moss-Morris R. Weinman J. Petrie K. J. Horne R. Cameron L. D. Buick D. (2002). The revised Illness Perception Questionnaire (IPQ-R). *Psychol. Health* 17:1. 10.1080/08870440290001494

[B55] Mubarak N. Zin C. S. (2020). Religious tourism and mass religious gatherings – the potential link in the spread of COVID-19. current perspective and future implications. *Travel Med. Infect. Dis.* 36:101786. 10.1016/j.tmaid.2020.101786 32531422 PMC7282735

[B56] New Straits Times (2020). *Covid-19 Challenge Looms as Churches, Mosques Re-open Worldwide.* Malaysia: New Straits Times.

[B57] Nisbett R. E. Miyamoto Y. (2005). The influence of culture: holistic versus analytic perception. *TCS* 9 467–473. 10.1016/j.tics.2005.08.004 16129648

[B58] Nurasikin M. S. Khatijah L. A. Aini A. Ramli M. Aida S. A. Zainal S. A. (2013). Religiousness, religious coping methods and distress level among psychiatric patients in Malaysia. *Int. J. Soc. Psychiatry* 59 332–338. 10.1177/0020764012437127 22408116

[B59] Oman D. Syme S. L. (2018). “Weighing the evidence: what is revealed by 100+ meta-analyses and systematic reviews of religion/spirituality and health?,” in *Why Religion and Spirituality Matter for Public Health*, ed. Oman D. (New York, NY: Springer International Publishing), 261. 10.1007/978-3-319-73966-3_15

[B60] Ozamix-Etxebarria N. Mondragon N. I. Santamaria M. D. Gorrotxategi M. P. (2020). Psychological symptoms during the two stages of lockdown in response to the COVID-19 outbreak: an investigation in a sample of citizens in northern Spain. *Front. Psychol.* 11:1491. 10.3389/fpsyg.2020.01491 32625157 PMC7314923

[B61] Pargament K. (1997). *The Psychology of Religion and Coping.* New York, NY: Guilford Press.

[B62] Pargament K. Feuille M. Burdzy D. (2011). The brief RCOPE: current psychometric status of a short measure of religious coping. *Religions* 2 51–76. 10.3390/rel2010051

[B63] Pargament K. I. Koenig H. G. Perez L. M. (2000). The many methods of religious coping: development and initial validation of the RCOPE. *J. Clin. Psychol.* 56 519–543. 10.1002/(sici)1097-4679(200004)56:4<519::aid-jclp6>3.0.co;2-1 10775045

[B64] Patton M. Q. (1990). *Qualitative Evaluation and Research Methods*, 2nd Edn. California: SAGE Publications.

[B65] Power L. McKinney C. (2013). The effects of religiosity on psychopathology in emerging adults: intrinsic versus extrinsic religiosity. *JORH* 53 1529–1538. 10.1007/s10943-013-9744-8 23771804

[B66] R Core Team (2013). *R: A Language and Environment for Statistical Computing.* Vienna: R Foundation for Statistical Computing.

[B67] Radhi N. A. M. (2020). *‘Wait Until MCO is Lifted’.* Malaysia: New Straits Times.

[B68] Rein R. (2016). *Meaning in Action: Outline of an Integral Theory of Culture.* Cambridge: Polity.

[B69] Roberti J. Harrington L. Storch E. (2006). Further psychometric support for the 10-item version of the perceived stress scale. *JCC* 9 135–147. 10.1002/j.2161-1882.2006.tb00100.x

[B70] Rodzi N. (2021). *Kuala Lumpur’s Usually Busy Streets Turn Quiet as Malaysia goes into full Covid-19 Lockdown.* Singapore: The Straits Times.

[B71] Roger K. S. Hatala A. (2017). Religion, spirituality and chronic illness: a scoping review and implications for health care practitioners. *J. Relig. Spirituality Soc. Work Soc. Thought* 37 24–44. 10.1080/15426432.2017.1386151

[B72] Rosenberg C. E. (1992). *Explaining Epidemics and other Studies in the History of Medicine.* Cambridge: Cambridge University Press.

[B73] Roser M. Ritchie H. Ortiz-Ospina E. Hasell J. (2021). *Malaysia: Coronavirus Pandemic Country Profile.* Available online at: https://ourworldindata.org/coronavirus/country/malaysia (accessed June 4, 2021).

[B74] Samuels J. (2017). “Contemporary Buddhism in Malaysia,” in *The Oxford Handbook of Contemporary Buddhism*, ed. Jerryson M. (Oxford: Oxford University Press), 10.1093/oxfordhb/9780199362387.013.1

[B75] Saroglou V. Cohen A. B. (2011). Psychology of culture and religion: introduction to the JCCP Special Issue. *J. Cross-Cult. Psychol.* 42 1309–1319. 10.1177/0022022111412254

[B76] Schulz J. Bahrami-Rad D. Beauchamp J. Henrich J. (2019). The church, intensive kinship, and global psychological variation. *Science* 366:eaau5141. 10.1126/science.aau5141 31699908

[B77] Shanmugam H. Juhari J. Nair P. Chow S. Ng C. (2020). Impacts of COVID-19 pandemic on mental health in Malaysia: a single thread of hope. *Malays J. Psychiatry* 29 78–84.

[B78] Shaw T. Ishak D. Lie D. Menon S. Courtney E. Li S. T. (2018). The influence of Malay cultural beliefs on breast cancer screening and genetic testing: a focus group study. *Psychooncology* 27 2855–2861. 10.1002/pon.4902 30264524

[B79] Sundararajan L. (2011). Spiritual transformation and emotion: a semiotic analysis. *J. Spiritual Ment. Health* 13 78–90. 10.1080/19349637.2011.547141

[B80] Sundararajan L. (2015). *Understanding Emotion in Chinese Culture.* New York, NY: Springer International Publishing, 10.1007/978-3-319-18221-6

[B81] Sundararajan L. (2020). Strong-ties and weak-ties rationalities: toward an expanded network theory. *Rev. Gen. Psychol.* 24 134–143. 10.1177/1089268020916438

[B82] Talhelm T. English S. (2020). Historically rice-farming societies have tighter social norms in China and worldwide. *PNAS* 117 19816–19824. 10.1073/pnas.1909909117 32732432 PMC7443949

[B83] Tan M. M. Su T. T. Ting R. S. K. Allotey P. Reidpath D. (2020). Religion and mental health among older adults: ethnic differences in Malaysia. *Aging Ment. Health* 10.1080/13607863.2020.1799939 Online ahead of print. 32741203

[B84] Tan M. M. Musa A. F. Su T. T. (2021). The role of religion in mitigating the COVID-19 pandemic: the Malaysian multi-faith perspectives. *Health Promot. Int.* 2021:daab041. 10.1093/heapro/daab041 33928389 PMC8135627

[B85] Tang C. P. (2015). The diversity of Malaysian Chinese Buddhism. *JCLC* 3 32–50.

[B86] Tartaro J. Luecken L. Gunn H. (2005). Exploring heart and soul: effects of religiosity/spirituality and gender on blood pressure and cortisol stress responses. *J. Health Psychol.* 10 753–766. 10.1177/1359105305057311 16176954

[B87] Taylor J. (2015). Psychometric analysis of the ten-item perceived stress scale. *Psychol. Assess.* 27 90–101. 10.1037/a0038100 25346996

[B88] The Star (2020a). *57% Increase in Domestic Violence Calls During MCO not True, Ministry Refutes Report.* Malaysia: The Star.

[B89] The Star (2020b). *Ghostly Going-on: Malaysia Phantom on Lockdown Patrol.* Malaysia: The Star.

[B90] The Straits Times (2020). *Malaysia to Allow Mass Prayers Limited to 30 People Ahead of Hari Raya Puasa as Coronavirus Cases Fall.* Singapore: The Straits Times.

[B91] The Sun Daily (2020). *MCO Phase 3: What’s New, Challenges Faced, and What Lies Ahead.* Malaysia: The Sun Daily.

[B92] Thomas J. Barbato M. (2020). Positive religious coping and mental health among Christians and Muslims in response to the COVID-19 pandemic. *Religions* 11:498. 10.3390/rel11100498

[B93] Ting R. S. K. Watson T. (2007). Is suffering good? An explorative study on the religious persecution of Chinese pastors. *J. Psychol. Theol.* 35 202–210.

[B94] Ting L. W. Perimbanayagam K. (2020). *Reopening of Temples, Churches will be Gradual.* Malaysia: New Straits Times.

[B95] Ting R. S. K. Mah A. S. C. Zhang K. J. (2020). “Chinese traditional religions and mental health: an indigenous psychology perspective,” in *The Psychology of World Religions and Spiritualities: An Indigenous Perspective*, eds Sisemore T. A. Knabb J. J. (Pennsylvania, PA: Templeton Press), 237–262.

[B96] Ting R. S. K. Ng A. (2012). Use of religious resources in psychotherapy from a tradition-sensitive approach: cases from Chinese in Malaysia. *Pastoral Psychol.* 61 941–957. 10.1007/s11089-011-0365-4

[B97] Ting R. S. K. Sundararajan L. (2018). *Culture, Cognition, and Emotion in China’s Religious Ethnic Minorities: Voices of Suffering Among the Yi.* Switzerland: Palgrave Macmillan.

[B98] Ting R. S. K. Sundararajan L. Huang Q. (2017). Narratives of suffering: a psycholinguistic analysis of two Yi religious communities in southwest China. *RSSR* 28 231–254. 10.1163/9789004348936_012 23104645

[B99] Ting R. S. K. Zhang K. J. Huang Q. B. (2019). “Indigenous psychology for all Chinese: heeding the mind and spirit of ethnic minorities in China,” in *Asian Indigenous Psychologies in Global Context*, ed. Yeh K. H. (Switzerland: Palgrave MacMillan), 249–276. 10.1007/978-3-319-96232-0_11

[B100] Todd P. M. Gigerenzer G. (2012). *Ecological Rationality: Intelligence in the World.* Oxford: Oxford University Press, 10.1093/acprof:oso/9780195315448.001.0001

[B101] Togoh I. (2020). *U.N. Chief Urges Governments: ‘Prioritise Women’s Safety’ as Domestic Abuse Surges During Coronavirus Lockdowns.* New Jersey, NJ: Forbes.

[B102] Tucker B. (2013). “Cultural ecology,” in *Theory in Social and Cultural Anthropology: an Encyclopedia*, eds McGee R. J. Warms R. L. (California: SAGE Publications), 142–147.

[B103] Vaughn P. Turner C. (2016). Decoding via coding: analyzing qualitative text data through thematic coding and survey methodologies. *J. Libr. Admin.* 56 41–51. 10.1080/01930826.2015.1105035

[B104] Villani D. Sorgente A. Iannello P. Antonietti A. (2019). The role of spirituality and religiosity in subjective well-being of individuals with different religious status. *Front. Psychol.* 10:1525. 10.3389/fpsyg.2019.01525 31354566 PMC6630357

[B105] Wang X. M. (2016). “The Chinese language in the Asian diaspora: a Malaysian experience,” in *Communicating With Asia: The Future of English as a Global Language*, eds Leitner G. Hashim A. Wolf H.-G. (Cambridge: Cambridge University Press), 205–215. 10.1017/cbo9781107477186.014

[B106] Weber S. Pargament K. (2014). The role of religion and spirituality in mental health. *Curr. Opin. Psychiatry* 27 358–363. 10.1097/yco.0000000000000080 25046080

[B107] World Health Organization [WHO] (2020a). *COVID-19 in Malaysia Situation Report 20.* Geneva: WHO.

[B108] World Health Organization [WHO] (2020b). *WHO COVID-19 Dashboard.* Geneva: WHO.

[B109] Yap C. K. Ting R. S. K. Tan L. T. N. Aw Yong Y. Y. Tan M. M. (2021). *Religion, Illness Perception and Coping in Pandemics: a Systematic Review.* Nagpur: RSSSR.

[B110] Yap K. H. Warren N. Allotey P. Reidpath D. D. (2019). Understandings of stroke in rural Malaysia: ethnographic insights. *Disabil. Rehabil.* 43 345–353. 10.1080/09638288.2019.1624841 31169419

[B111] You S. Lim S. (2018). Religious orientation and subjective well-being: the mediating role of meaning in life. *JPT* 47 34–47. 10.1177/0091647118795180

[B112] Yusof T. A. (2021). *Malaysia Raises Mental Health and Social Well-being Concerns Amidst Pandemic.* Malaysia: New Straits Times.

[B113] Zhang M. Hong L. Zhang T. Lin Y. Zheng S. Zhou X. (2016). Illness perceptions and stress: mediators between disease severity and psychological well-being and quality of life among patients with Crohn’s disease. *Patient Prefer Adherence* 10 2387–2396. 10.2147/ppa.s118413 27920505 PMC5125764

