# Supplementary material for: Cultural Responses to Covid-19 Pandemic: Religions, Illness Perception, and Perceived Stress
Source: Front Psychol. 2021 Jul 23;12:634863. doi: 10.3389/fpsyg.2021.634863 (PMC8375556; doi:10.3389/fpsyg.2021.634863)
Supplement: Supplementary file 1 [file Table_1.docx]

***Supplementary Material***

# Supplementary Tables

**Supplementary Table 1.** Partial Correlations between Religious Expression and Perceived Stress after Controlling for All Illness Perception Domains

| Variables | 1 | 2 | 3 |
| --- | --- | --- | --- |
| 1. Perceived Stress total score | — |  |  |
| 1. External Religious Expression | -.10* | — |  |
| 1. Internal Religious Expression | -.12* | .77*** | — |

*Note*. **p* < .05. ****p* ≤ .001.

**Supplementary Table 2.** Partial Correlations between Illness Perception and Perceived Stress after Controlling for External and Internal Religious Expression

| Variables | 1 | 2 | 3 | 4 | 5 | 6 | 7 | 8 | 9 |
| --- | --- | --- | --- | --- | --- | --- | --- | --- | --- |
| 1. Perceived Stress total score | — |  |  |  |  |  |  |  |  |
| 1. (*Consequences*) How much does COVID-19 affect your life? | .28*** | — |  |  |  |  |  |  |  |
| 1. (*Timeline*) How long do you think the COVID-19 pandemic will continue? | .15*** | .15*** | — |  |  |  |  |  |  |
| 1. (*Personal control*) How much control do you feel you have over the COVID-19 pandemic? | -.16*** | -.07 | .03 | — |  |  |  |  |  |
| 1. (*Concern*) How concerned are you about the COVID-19 pandemic? | .07 | .29*** | .10* | .14*** | — |  |  |  |  |
| 1. (*Comprehensibility*) How well do you feel you understand the COVID-19 pandemic? | -.14*** | .02 | .07 | .24*** | .25*** | — |  |  |  |
| 1. (*Emotions*) How much does the COVID-19 pandemic affect you emotionally (e.g. does it make you angry, scared, upset or depressed)? | .49*** | .42*** | .15*** | -.03 | .27*** | .03 | — |  |  |
| 1. (*Severity*) How severe do you think of the COVID-19 as a disease? | .11** | .14*** | .18*** | .02 | .37*** | .21*** | .19*** | — |  |
| 1. (*Likelihood of Contracting*) How likely do you think you would contract the COVID-19? | .19*** | .15*** | .16*** | -.12** | .05 | -.04 | .18*** | .02 | — |

*Note*. **p* < .05. ***p* < .01. ****p* ≤ .001.

# Supplementary Table 3. Regression Coefficients for Illness Perception Domains Predicting Perceived Stress

| Variables | *b* [95% CI] | *SE* | *p* | *sr^2^* | *R^2^* | Adj. *R^2^* |
| --- | --- | --- | --- | --- | --- | --- |
|  |  |  |  |  | .316 | .306 |
| BIPQ Consequences | .245 | .115 | .033 | .005 |  |  |
| BIPQ Timeline | .327 | .141 | .021 | .006 |  |  |
| BIPQ Personal Control | -.280 | .097 | .004 | .010 |  |  |
| BIPQ Concern | -.207 | .131 | .115 | .003 |  |  |
| BIPQ Comprehensibility | -.585 | .154 | < .000 | .017 |  |  |
| BIPQ Emotions | 1.157 | .100 | < .000 | .154 |  |  |
| BIPQ Severity | .163 | .151 | .283 | .001 |  |  |
| BIPQ Likelihood of Contracting | .218 | .105 | .039 | .005 |  |  |

*Note*. CI = Confidence Interval. SE = Standard Error.

**Supplementary Table 4.** Comparison of Themes Frequency Between Different Religious Groups

|  | Buddhists | | Christians | | Muslims | | Total | |
| --- | --- | --- | --- | --- | --- | --- | --- | --- |
| Number of Responses | 712 | | 847 | | 282 | | 1841 | |
| Themes and Subthemes | *n* | *%* | *n* | *%* | *n* | *%* | *N* | % |
| Consequences of human behaviour | 203 | 28.51 | 244 | 28.81 | 107 | 37.94 | 554 | 30.09 |
| Poor public health behaviour | 112 | 15.73 | 132 | 15.58 | 59 | 20.92 | 292 | 15.86 |
| Unusual eating choices and behaviour | 37 | 5.2 | 29 | 3.42 | 24 | 8.51 | 90 | 4.89 |
| Public fail to follow government protocols | 23 | 3.23 | 23 | 2.72 | 15 | 5.32 | 72 | 3.91 |
| Environment disaster caused by humans | 6 | 0.84 | 14 | 1.65 | 4 | 1.42 | 24 | 1.30 |
| Uncooperative community | 9 | 1.26 | 13 | 1.53 | 0 | 0 | 22 | 1.20 |
| High risk individuals not following protocols | 6 | 0.84 | 12 | 1.42 | 1 | 0.35 | 19 | 1.03 |
| Unhealthy lifestyle | 3 | 0.42 | 12 | 1.42 | 2 | 0.71 | 17 | 0.92 |
| Reckless behaviour | 5 | 0.7 | 7 | 0.83 | 0 | 0 | 12 | 0.65 |
| Fake news | 2 | 0.28 | 2 | 0.24 | 2 | 0.71 | 6 | 0.33 |
| Consequences of human attitudes | 194 | 27.25 | 225 | 26.56 | 51 | 18.09 | 470 | 25.53 |
| Lack of awareness and education | 74 | 10.39 | 64 | 7.56 | 16 | 5.67 | 154 | 8.37 |
| Human flawed characters | 36 | 5.06 | 62 | 7.32 | 9 | 3.19 | 107 | 5.81 |
| Human ignorance | 35 | 4.92 | 41 | 4.84 | 12 | 4.26 | 88 | 4.78 |
| Underestimated the severity of virus | 19 | 2.67 | 23 | 2.72 | 6 | 2.13 | 48 | 2.61 |
| Public mindset | 18 | 2.53 | 16 | 1.89 | 4 | 1.42 | 38 | 2.06 |
| Human attitudes | 6 | 0.84 | 15 | 1.77 | 2 | 0.71 | 23 | 1.25 |
| Public emotional reaction | 6 | 0.84 | 4 | 0.47 | 2 | 0.71 | 12 | 0.65 |
| Socio-political reasons | 95 | 13.34 | 116 | 13.69 | 39 | 13.83 | 250 | 13.58 |
| Ineffective government | 44 | 6.18 | 43 | 5.08 | 16 | 5.67 | 103 | 5.59 |
| Man-made disaster | 10 | 1.4 | 19 | 2.24 | 5 | 1.77 | 34 | 1.85 |
| Poor medical resources | 19 | 2.67 | 10 | 1.18 | 3 | 1.06 | 32 | 1.74 |
| Poor preventive measures | 9 | 1.26 | 12 | 1.42 | 1 | 0.35 | 22 | 1.20 |
| Caused by China | 3 | 0.42 | 10 | 1.18 | 7 | 2.48 | 20 | 1.09 |
| Economical factor | 6 | 0.84 | 9 | 1.06 | 5 | 1.77 | 20 | 1.09 |
| International politics | 4 | 0.56 | 11 | 1.3 | 1 | 0.35 | 16 | 0.87 |
| Caused by USA | 0 | 0 | 2 | 0.24 | 1 | 0.35 | 3 | 0.16 |
| Social factors | 108 | 15.17 | 79 | 9.33 | 41 | 14.54 | 228 | 12.38 |
| Social gathering | 41 | 5.76 | 17 | 2.01 | 11 | 3.9 | 69 | 3.75 |
| Human interaction | 24 | 3.37 | 26 | 3.07 | 10 | 3.55 | 60 | 3.26 |
| Human mobility | 12 | 1.69 | 11 | 1.3 | 13 | 4.61 | 36 | 1.96 |
| Human existence | 15 | 2.11 | 9 | 1.06 | 4 | 1.42 | 28 | 1.52 |
| Space sharing | 6 | 0.84 | 9 | 1.06 | 3 | 1.06 | 18 | 0.98 |
| Religious factor | 8 | 1.12 | 5 | 0.59 | 0 | 0 | 13 | 0.71 |
| Cultural factor | 2 | 0.28 | 2 | 0.24 | 0 | 0 | 4 | 0.22 |
| Medical explanation | 69 | 9.69 | 114 | 13.46 | 24 | 8.51 | 207 | 11.24 |
| Poor immune system | 21 | 2.95 | 29 | 3.42 | 7 | 2.48 | 57 | 3.10 |
| Virus transmission | 17 | 2.39 | 24 | 2.83 | 3 | 1.06 | 44 | 2.39 |
| Infections | 10 | 1.4 | 15 | 1.77 | 5 | 1.77 | 30 | 1.63 |
| Bio-mutation | 4 | 0.56 | 16 | 1.89 | 5 | 1.77 | 25 | 1.36 |
| Contact with COVID-19 positive cases | 6 | 0.84 | 10 | 1.18 | 0 | 0 | 16 | 0.87 |
| Physical contact | 2 | 0.28 | 8 | 0.94 | 2 | 0.71 | 12 | 0.65 |
| Hard to detect | 3 | 0.42 | 5 | 0.59 | 1 | 0.35 | 9 | 0.49 |
| Physical symptoms | 3 | 0.42 | 4 | 0.47 | 1 | 0.35 | 8 | 0.43 |
| High risk and severity rate | 2 | 0.28 | 2 | 0.24 | 0 | 0 | 4 | 0.22 |
| Microbiology transmission | 1 | 0.14 | 1 | 0.12 | 0 | 0 | 2 | 0.11 |
| Ecological explanation | 37 | 5.2 | 39 | 4.61 | 12 | 4.26 | 88 | 4.78 |
| Environment problem | 18 | 2.53 | 18 | 2.13 | 5 | 1.77 | 41 | 2.23 |
| Natural process | 9 | 1.26 | 9 | 1.06 | 0 | 0 | 18 | 0.98 |
| Animal contact | 2 | 0.28 | 6 | 0.71 | 6 | 2.13 | 14 | 0.76 |
| Population problem | 6 | 0.84 | 5 | 0.59 | 1 | 0.35 | 12 | 0.65 |
| Natural disaster | 2 | 0.28 | 1 | 0.12 | 0 | 0 | 3 | 0.16 |
| Religious-spiritual explanation | 6 | 0.84 | 30 | 3.54 | 8 | 2.84 | 44 | 2.39 |
| Karma/Sin | 3 | 0.42 | 9 | 1.06 | 0 | 0 | 12 | 0.65 |
| Will of God | 2 | 0.28 | 5 | 0.59 | 4 | 1.42 | 11 | 0.60 |
| Punishment from God | 1 | 0.14 | 8 | 0.94 | 1 | 0.35 | 10 | 0.54 |
| Fatalism | 0 | 0 | 2 | 0.24 | 3 | 1.06 | 5 | 0.27 |
| Testing from God | 0 | 0 | 3 | 0.35 | 0 | 0 | 3 | 0.16 |
| Personal faith | 0 | 0 | 3 | 0.35 | 0 | 0 | 3 | 0.16 |
